# The influence of insecticide exposure and environmental stimuli on the movement behaviour and dispersal of a freshwater isopod

**DOI:** 10.1007/s10646-016-1686-y

**Published:** 2016-06-15

**Authors:** Jacqueline Augusiak, Paul J. Van den Brink

**Affiliations:** 1 Aquatic Ecology and Water Quality Management Group, Wageningen University and Research centre, P.O. Box 47, 6700 AA Wageningen, The Netherlands; 2 Alterra, Wageningen University and Research centre, P.O. Box 47, 6700 AA Wageningen, The Netherlands

**Keywords:** Locomotion, Dispersal, Automated video tracking, Aquatic macroinvertebrates

## Abstract

Behaviour links physiological function with ecological processes and can be very sensitive towards environmental stimuli and chemical exposure. As such, behavioural indicators of toxicity are well suited for assessing impacts of pesticides at sublethal concentrations found in the environment. Recent developments in video-tracking technologies offer the possibility of quantifying behavioural patterns, particularly locomotion, which in general has not been studied and understood very well for aquatic macroinvertebrates to date. In this study, we aim to determine the potential effects of exposure to two neurotoxic pesticides with different modes of action at different concentrations (chlorpyrifos and imidacloprid) on the locomotion behaviour of the water louse *Asellus aquaticus*. We compare the effects of the different exposure regimes on the behaviour of *Asellus* with the effects that the presence of food and shelter exhibit to estimate the ecological relevance of behavioural changes. We found that sublethal pesticide exposure reduced dispersal distances compared to controls, whereby exposure to chlorpyrifos affected not only animal activity but also step lengths while imidacloprid only slightly affected step lengths. The presence of natural cues such as food or shelter induced only minor changes in behaviour, which hardly translated to changes in dispersal potential. These findings illustrate that behaviour can serve as a sensitive endpoint in toxicity assessments. However, under natural conditions, depending on the exposure concentration, the actual impacts might be outweighed by environmental conditions that an organism is subjected to. It is, therefore, of importance that the assessment of toxicity on behaviour is done under relevant environmental conditions.

**Electronic supplementary material:**

The online version of this article (doi:10.1007/s10646-016-1686-y) contains supplementary material, which is available to authorized users.

## Introduction

Arthropod populations form an integral part of freshwater ecosystems and are, as such, often exposed to chemical and physical disturbances such as nutrients, pollutants, habitat destruction and flow alterations (Dudgeon et al. 2006). In agro-ecosystems, pesticides used for plant protection in particular can enter surface waters through spray drift, run off, and draining, and affect non-target animal populations. Hence, environmental risk assessments are required for pesticides to minimize undesired side effects. Standard tests comprise a battery of mortality, immobilization and reproduction studies on single species in the lower tiers of the assessment process. In the higher tiers, micro- and mesocosms may be employed to evaluate ecological community responses to different exposure concentrations (Brock et al. 2006).

To improve the determination of ecologically relevant risk levels, behavioural endpoints are increasingly investigated in ecotoxicological studies (Rodrigues et al. 2016). They have been shown to be relevant and useful in acute and chronic environmental risk assessments because they link physiological functions with ecological processes. Behavioural endpoints are also very sensitive towards environmental stimuli and chemical exposure (Dell’Omo 2002), and several studies assessing the environmental risks of pesticides reported behavioural effects at concentrations significantly below those causing mortality (for examples see Böttger et al. 2013; Agatz et al. 2014). Locomotor behaviour is particularly vital to animal life as it facilitates feeding, predator avoidance, reproduction, or migration, and thus may link the effects of individual stress to the population level (Bayley et al. 1997). This type of behaviour can be studied easily via video tracking (Augusiak and Van den Brink 2015; Rodrigues et al. 2016).

In aquatic environments, relocating macroinvertebrates are likely to encounter contaminated stretches with residue concentrations of pesticides. Depending on the mode of action and concentration of the encountered pesticide, travelling animals may be affected and their movement behaviour may be likely to change under such conditions. Especially neurotoxic substances might adversely affect orientation and activity. The observed alterations in activity, furthermore, correlated with the measured contamination gradient. Baatrup and Bayley (1993) showed that cypermethrin exposure disrupted the general movement pattern and activity of the Wolf Spider *Pardosa amentata*. However, studies on the behavioural effect of toxicants on aquatic crustaceans, so far mainly focused on feeding responses (Böttger et al. 2013; Agatz et al. 2014), induction of drift (Beketov and Liess 2008), breathing activity, and immobilization (for example Rubach et al. 2011). Fewer studies attempted quantification of more complex behaviour such as precopulatory mate guarding (Blockwell et al. 1998) or predator–prey interactions (Brooks et al. 2009) after sublethal pesticide exposure. To estimate the impact of chemical exposure on arthropod populations in an ecologically more meaningful way, ecological effect models are increasingly often applied to integrate different habitat, species, and exposure related information to assess population recovery timeframes (Galic et al. 2013; Focks et al. 2014). Accounting for immigrating and emigrating individuals is essential to improve the mechanistic understanding derived from such modelling studies (Focks et al. 2014; Hommen et al. 2015).

With the present study, we present a method to test the effects of chemical exposure on macroinvertebrate movement, and to improve the understanding of the potential effects of exposure to neurotoxic pesticides, in this case chlorpyrifos and imidacloprid, on the water louse *Asellus aquaticus*. To establish a broader knowledge of the background levels and variance of the movement responses we included observations of non-exposed specimens under environmentally relevant scenarios such as the presence or absence of food and shelter items.

Imidacloprid is a selective and systemic insecticide belonging to the group of neonicotinoids that agonistically affect nicotinic acetylcholine receptors (nAChRs) of insects (Matsuda et al. 2001). Chlorpyrifos, on the other hand, is an organophosphate insecticide that inhibits acetylcholine esterase, which is essential to nerve function in insects, humans, and other animals (Pope 2010), thus acting as a broad-spectrum agent (Song et al. 1997). Exposure to either substance, however, can eventually cause paralysis and death. We aimed to test whether the differences in mode of action would lead to different effects on the locomotion behaviour and whether the responses are concentration-dependent.

*A*. *aquaticus* is widely distributed throughout Europe, and is relatively sensitive to insecticides (Wogram and Liess 2001). As consumers at an intermediate trophic level, they also fulfil an important role in the nutrient cycling of aquatic ecosystems (Wallace and Webster 1996). Their population recovery processes are limited since the species has a fully aquatic life-cycle with virtually no possibility to reoccupy exposed patches by air. Recovery, hence, depends mostly on the intrinsic reproduction potential and dispersal of individuals within a water body from uncontaminated patches towards exposed ones. This species also appeared to be easily studied using automated video tracking (Augusiak and Van den Brink 2015).

## Materials and methods

### Test species

Adult *A*. *aquaticus* were collected from a non-contaminated pond (Duno pond, Doorwerth, The Netherlands) with sweeping nets, and organisms larger than approximately 5 mm were transferred to the laboratory. The specimens were kept in a 30 L aquarium in a climate-controlled room at 18 °C and a 10:14 light:dark cycle. Prior to the experiments, the organisms were acclimatised to copper-free water over 1 week by a sequential diluting process of the original pond water with copper-free water. Dried poplar leaves were provided as food source ad libitum and aeration was constantly supplied. Individuals for the experiments were chosen randomly from this stock (mean body length ± standard deviation: 6.4 mm ± 0.66).

### Experimental setup

The movement observations were performed in a climate-controlled room at 20 °C. The test setup consisted of a camera mounted above an aquarium of 1 m^2^, which was filled with a 0.5 cm layer of quartz sand and 10 cm of copper free tap water. Before the observations, individual specimens were marked with rectangular paper snippets of approximately 2 × 2 mm, left for 1 h to recover from the marking procedure, and introduced into the aquarium. Small droplets of cyanoacrylate (Pattex, Gold Gel) were used to fix the marker to the backs of the *Asellus*. After introduction into the aquarium and 30 min acclimation time, animal movements were recorded for 1 h and the tracks statistically evaluated to determine movement related parameters. We used a digital single-lens reflex camera (EOS 1100D, Canon) for the recordings, which was connected to a computer. Four of such aquarium-camera combinations were installed in parallel within a water bath that maintained constant temperatures. See Augusiak and Van den Brink (2015) for further details about the used methodology.

Water temperature, pH and dissolved oxygen were measured twice every day to ascertain stable conditions throughout the experimental period. All experiments were carried out at a water temperature of 20 ± 0.8 °C, an average pH of 7.6 ± 0.3 (measured with electrode pH323, WTW Germany) and an average dissolved oxygen level of 8.6 ± 0.3 mg/L (measured with oximeter Oxi330 equipped with sensor CellOx 325, WTW Germany).

### Test chemicals: application, sampling, and analysis

Exposure concentrations were derived from toxicity tests performed prior to the behavioural study (see Online Resource 1 for details). Solutions of chlorpyrifos were prepared by spiking copper-free water with an aqueous stock solution of chlorpyrifos (480 g/L) to reach exposure concentrations of 0, 0.6 and 1.5 μg/L (48 h-EC50 = 3.2 μg/L, 48 h-EC10 = 2.7 μg/L, Online Resource 1).

Water samples from the controls and exposure vessels were taken at the start and after 48 h of exposure to confirm concentrations. In the beginning, 200 mL samples were taken from the spiked batch volume; at the end, 200 mL per exposure vessel were sampled. Chlorpyrifos was measured by liquid–liquid extraction with 20 mL n-hexane followed by gas chromatography coupled with electron capture detection (GC-ECD). The specifications for the sample analysis via GC-ECD were in accordance with the study by Rubach et al. (2011).

Dosing solutions of imidacloprid were prepared by mixing a soluble formulation containing 200 g imidacloprid/L into copper-free water, yielding an 80 ppm stock solution, which was used to spike the exposure solutions of 0, 37.5 and 75 μg/L (48 h-EC50 = 603 μg/L, 48 h-EC10 = 225 μg/L, Online Resource 1). Water samples from the controls and exposure vessels were taken at the start and after 48 h of exposure to confirm concentrations. For this, samples of approximately 3 mL were transferred into 4 mL glass vials that contained 1 mL acetonitrile. After mixing, the vials were stored at −20 °C prior to analysis. Specifications for the water sample analysis via liquid chromatography–tandem mass spectrometry (LC–MS/MS) were analogous to the study by Roessink et al. (2013).

### Test conditions

To study the effects of sublethal pesticide exposure on the dispersal behaviour, specimens were exposed to the respective pesticide concentration for 48 h prior to the marking and video observation procedure. After 48 h, the animals were removed from the exposure vessels and transferred into clean, copper-free tap water. Water quality parameters were measured in the beginning and the end of the exposure phase and water samples taken for chemical analysis at the same time. During the chlorpyrifos exposure, the water temperature was 20.1 ± 1.6 °C, the average pH was 6.8 ± 0.8 (measured with electrode pH323, WTW Germany) and the average dissolved oxygen level was 7.9 ± 0.2 mg/L (measured with oximeter Oxi330 equipped with sensor CellOx 325, WTW Germany). During the imidacloprid exposure the water temperature was 20.0 ± 1.4 °C, the average pH 7.8 ± 0.2 and the average dissolved oxygen level was 7.5 ± 1.2 mg/L. Control groups were kept under similar conditions, except that no pesticide was added.

To test the effect of potential food items being present, we cut leaves found in the animals’ native environment into 5 × 5 cm rectangular pieces and hung four such fragments at evenly distributed spots into the water in the arenas. We used simple threads to fix the leaves and adjusted the vertical position in the water phase so that the leaf material was just immersed. Shelter experiments, on the other hand, were conducted with 5 × 10 cm big rectangles of stainless steel mesh wire structures that were placed at six evenly distributed spots in each arena. Control groups were handled similarly, except that no items were added to the arena. All experiments were conducted with two population densities, one and fifty individuals per arena, respectively, and were replicated twenty times each (Augusiak and Van den Brink 2015).

### Data analysis

We used the open source software ImageJ (Abramoff et al. 2004) to extract animal tracks from the recorded movies. Tracks within a 10 cm margin of the arena’s walls were dismissed to exclude potential bias due to edge behaviour (Creed and Miller 1990). The obtained time series of (x, y)-coordinates of the animals’ positions were analysed using the R software (R Core Team 2013) and the package “adehabitatLT” (Calenge 2006).

**Fig. 1:**
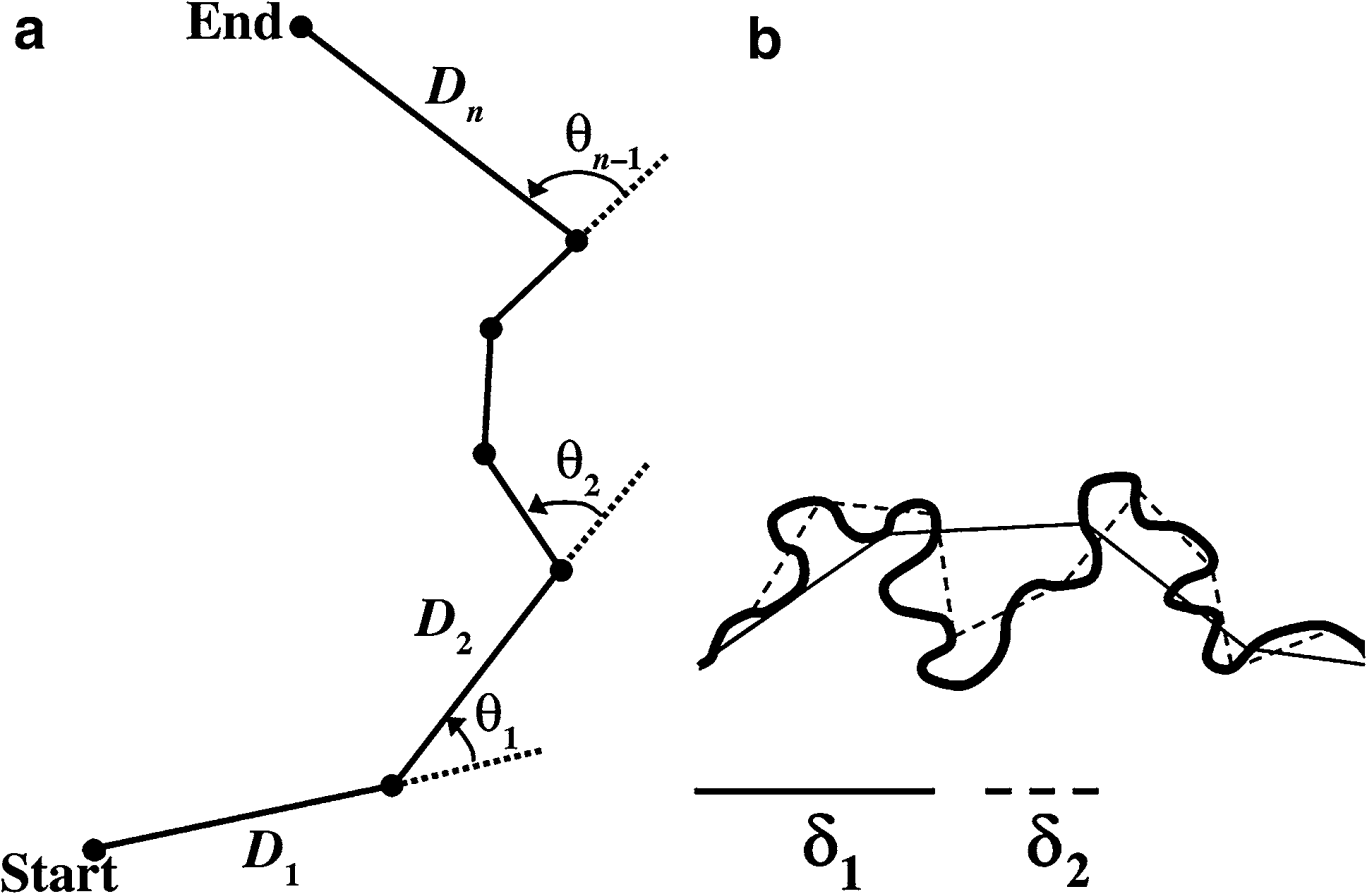
**a** Illustration of the components of a movement path. *Solid lines* represent the distance D_i_ travelled per time interval (*step length*). The *dashed lines* indicate the turning *angle* (θ) as the deviation from straight-line locomotion measured in degrees (±180°). **b** Schematic of the divider method. Two steps of the analysis are shown, using two different divider lengths δ (Adapted from Seuront et al. 2004a, b)

We defined relocations of less than 1 mm as resting moments (Augusiak and Van den Brink 2015), and calculated resting time per individual as the percentage time that the respective individual spent not moving. During periods of activity, behaviour was further characterized by step lengths and turning angles. Step length is defined as the distance covered per time interval, whereas angles between successive moves were measured as deviation from straight locomotion in degrees (±180°) (see Fig. 1a for a schematic representation of the path components). Since these metrics depend on the physical or temporal scale at which they are measured, we chose to further calculate the fractal dimension of each individual’s path. The fractal dimension is a measure of a path’s tortuosity and quantifies an object’s ability to cover the Euclidian space through which it navigates scale-independently (Seuront et al. 2004b). The parameter values range between *D* = 1 (straight line) to *D* = 2 (Brownian motion). We used the Fractal Mean Estimator contained in the Fractal software made available by Nams (1996) to calculate the fractal dimension for each path. If multiple paths were obtained for one individual, a mean value was estimated. The software makes use of the divider method (Mandelbrot 1967) and calculates the trajectory length (*L*) over a range of divider sizes (δ; see Fig. 1b for a schematic illustration) such that

$$L\left(\updelta \right) = {\text{k}}\updelta^{1 - D}$$

where *k* is constant, and *D* the fractal dimension of the trajectory. The fractal dimension can be calculated from a subsequent regression of log(*L*) as a function of log(δ). We used 200 divider sizes (δ) ranging from approximately half of a species’ body size (*Asellus*: 0.25 cm) to the observation scale of 100 cm. Movement tracks shorter than 5 relocation points were excluded from the estimation of fractal dimension values to facilitate a robust regression. For consistency among compared parameters, we limited the remaining data analysis to the same range.

The assumption of normality was violated for all variables, except a transformed version of the fractal dimension [log(*D*-1) transformed], restricting us to mostly non-parametric tests to assess differences between experimental conditions. Wilcoxon’s rank sum tests were applied to test for pairwise differences of resting times and step lengths between treatments, Kruskal–Wallis tests were used for comparing more than two treatments. To determine differences between fractal dimension values, we used the Welch’s *t* test, or in case of comparing more than two treatments, ANOVA. Standard methods of circular statistics were used to analyse the turning angles. Since the angular distributions exhibited varying concentration parameters κ, we used the non-parametric Watson–Wheeler test to compare treatment effects (Batschelet 1981). Significances were assessed at a 95 % confidence level.

The paths recorded under different experimental conditions were further analysed for deviances with a correlated random walk (CRW) model following the steps laid out in Turchin (1998). This type of model is suitable for evaluating paths in homogeneous environments and can be used to estimate the population dispersal rate within the respective substrate (Turchin 1998). For an analysis of movement paths according to the CRW model framework, a series of statistical approaches needs to be applied to test whether model assumptions are met.

The primary assumption in CRW models is that the organisms exhibit some degree of directional persistence, i.e. the stronger the directional persistence, the faster the population is assumed to spread. This can be checked visually via the frequency distribution of observed turning angles. CRW models furthermore assume that step lengths and turning angles within a path are not serially correlated (Turchin 1998). Such correlations can influence the model output and need to be interpreted accordingly (Turchin 1998; Westerberg et al. 2008; Dray et al. 2010). Auto-correlation for step-length and turning angles was estimated according to the procedures defined by Dray et al. (2010). The correlation between the magnitude of turning angles and step length was estimated using Spearman’s correlation.

For verifying the applicability of the CRW formulation, net-squared displacements (R_n_^2^) were calculated and comparisons made between estimated (theoretical) and observed (actual) values. Observed net-squared displacements were calculated as the squared distance between each location in an individual’s track and the individual’s original location. Directional information thereby is removed by using the square of the distances. According to the CRW framework, R_n_^2^ can be estimated and extrapolated as follows:

$$R_{n}^{2} = nL_{2} + 2L_{1}^{2} \frac{c}{1 - c}\left( {n - \frac{{1 - c^{n} }}{1 - c}} \right)$$

where L_1_ is the mean move length (cm), L_2_ is the mean squared move length (cm^2^), n is the number of consecutive moves, and c is the mean cosine of turning angles (Kareiva and Shigesada 1983; Turchin 1998). The 95 % confidence interval for the estimated R_n_^2^ was constructed following a procedure described by Turchin (1998).

## Results

Due to excluding short tracks and tracks within the outer 10 cm margin of the aquaria from the data analysis, we did not obtain tracking information for all time points. The number of data points analysed for each test regime along with the number of paths and their average duration are summarised in Table 1. Furthermore, Table 1 lists the intended and measured concentrations of the two studied pesticides. The achieved chlorpyrifos concentrations were approximately 40 % below the intended levels at the start of the exposure phase. During the course of the exposure the concentrations dropped due to evaporation, chemical degradation, and sorption processes. However, the concentration difference remained at a factor of approximately 2 between the higher and the lower concentration treatments, indicating that observed changes in behaviour were still comparable among the different exposures. Achieved imidacloprid concentrations, on the other hand, were slightly above the intended levels, with concentrations decreasing less strongly as in the case of chlorpyrifos.

**Table 1:** Basic path information and mean values of movement parameters estimated for the different experimental regimes with *A*. *aquaticus*

Density	Chlorpyrifos low (0.6 μg/L)		Chlorpyrifos high (1.5 μg/L)		Imidacloprid low (37.5 μg/L)		Imidacloprid high (75 μg/L)		Control (starved)		Control (fed)		Food		Shelter	
	1	50	1	50	1	50	1	50	1	50	1	50	1	50	1	50
Available data points (Percentage of total recording time)	29,760 41 %	33,098 46 %	28,132 39 %	31,668 44 %	11,295 17 %	27,450 38 %	21,432 30 %	23,127 32 %	19,484 27 %	23,212 32 %	27,807 39 %	23,263 32 %	26,189 36 %	25,569 36 %	11,291 16 %	12,119 17 %
Number of available paths	256	384	244	421	336	330	379	394	336	448	328	375	314	289	176	186
Average path duration (sec ± SD)	114.4 (±138.7)	85.2 (±139.2)	113.8 (±156.7)	74.2 (±96.2)	35.4 (±56.8)	81.9 (±143.0)	55.1 (±117.4)	57.4 (±105.3)	56.7 (±99.1)	50.8 (±85.9)	83.8 (±117.8)	60.9 (±94.2)	82.4 (±132.7)	87.2 (±136.8)	62.4 (±97.0)	63.1 (±92.2)
Average measured concentrations (t_0h_, t_48h_; μg/L ± SD)	0.40, 0.28 (±0.03, ± 0.06)		0.83, 0.75 (±0.05, ± 0.21)		42.09, 40.67 (±3.80, ± 3.94)		80.82, 77.61 (±2.80, ± 3.38)		–		–		–		–	
Resting time (±SD)	51.5 % (±26.7)	53.8 % (±29.4)	56.7 % (±29.9)	44.4 % (±21.4)	28.4 % (±16.7)	37.9 % (±24.5)	36.3 % (±27.4)	35.4 % (±22.6)	29.5 % (±10.3)	31.2 % (±15.9)	30.2 % (±12.4)	40.2 % (±13.7)	35.7 % (±13.5)	45.4 % (±19.1)	44.2 % (±19.4)	44.2 % (±11.0)
Step length (cm/sec ± SD)	0.79 (±0.37)	0.71 (±0.36)	0.53 (±0.31)	0.75 (±0.28)	0.82 (±0.30)	0.81 (±0.33)	0.74 (±0.42)	0.92 (±0.36)	1.12 (±0.25)	1.13 (±0.29)	0.99 (±0.25)	0.86 (±0.25)	0.94 (±0.25)	0.80 (±0.29)	0.86 (±0.32)	0.69 (±0.28)
Turning angle	1.55° (±28.10)	−0.91° (±35.41)	0.93° (±44.87)	1.19° (±32.85)	1.14° (±37.00)	3.74° (±37.19)	−1.57° (±45.18)	−0.15° (±43.20)	2.92° (±25.09)	−2.73° (±26.31)	2.56° (±27.77)	0.09° (±36.71)	−4.35° (±28.25)	−6.70° (±34.21)	−1.48° (±28.33)	0.15° (±38.57)
Fractal D (±SD)	1.14 (±0.18)	1.12 (±0.08)	1.30 (±0.26)	1.11 (±0.09)	1.24 (±0.18)	1.29 (±0.28)	1.17 (±0.13)	1.10 (±0.13)	1.16 (±0.17)	1.19 (±0.19)	1.23 (±0.26)	1.25 (±0.31)	1.18 (±0.19)	1.17 (±0.19)	1.10 (±0.11)	1.11 (±0.06)

### Observed movement and dispersal

**Fig. 2:**
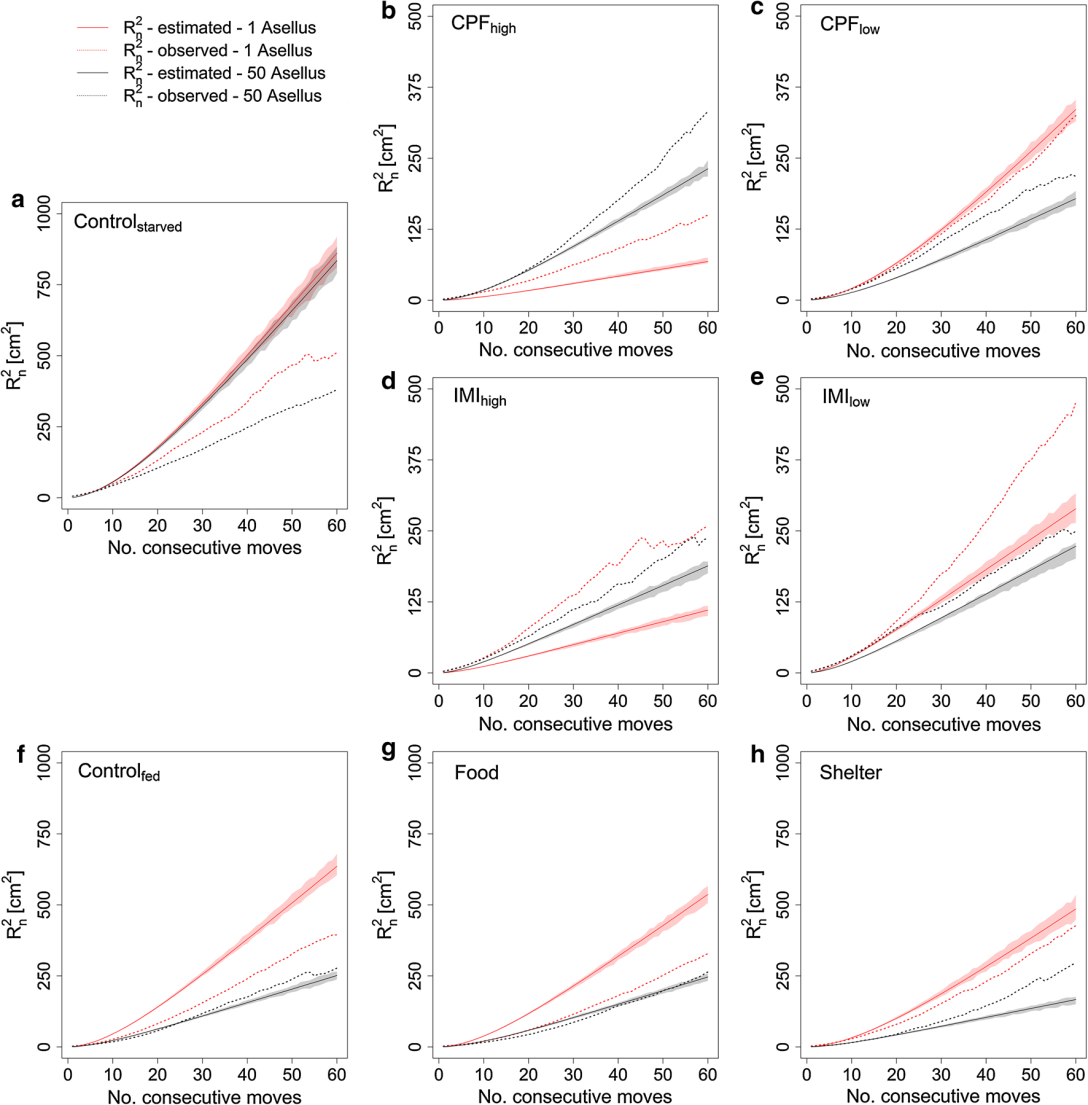
Relationship between the mean net-squared displacement (R_n_^2^; cm^2^) and the number of consecutive moves made by *A*. *aquaticus* under different experimental conditions. *Doted lines*: observed mean net-squared displacement obtained by averaging over 20 observed individuals; *dashed lines*: estimated net-squared displacement obtained by applying the observed average move distances and turning angles; *solid*: 95 % confidence interval of the estimated net-squared displacement; *red* stands for the single-*Asellus* studies and *black* for the 50-*Asellus* studies (Color figure online)

In Fig. 2 the relationship between the observed net-squared displacements (R_n_^2^) of *A*. *aquaticus* under different testing conditions and the number of consecutive steps they have made is represented with dashed lines. Net-squared displacement describes the ability of an organism to disperse, i.e. the smaller its value the closer an individual is to its original location. An individual’s R_n_^2^ over time is influenced by the combination of step lengths and turning angles it uses. The more active an animal is and the longer and more directed its subsequent steps are, the faster it will move away from its original location.

#### Pesticide exposure

Observed net-squared displacements were reduced by pesticide exposure compared to the respective controls (Fig. 2a–e). Higher exposure concentrations thereby caused stronger decreases in R_n_^2^ for both substances, except for the application of the higher chlorpyrifos dosage in the higher density setup. That treatment also changed the observed pattern of single individuals dispersing farther than their counterparts in a group (Fig. 2b). Compared to the controls, chlorpyrifos exposure increased resting times and decreased step lengths more than imidacloprid exposure did. The standard deviations of either parameter also increased but were, irrespective of the substance, concentration, or population density, overall in a more similar range than the mean values (Table 1). The control group exhibited slightly bigger average turning angles with lower variability than the exposed groups did, which however hardly affected the fractal dimension of the analysed paths. Resting times were affected significantly for all single-specimen observations, while step lengths were affected significantly or marginally significantly for both single- and 50-specimens observations (Table 2). Chlorpyrifos exposure had an overall statistically more significant effect on those parameters than imidacloprid exposure had. Turning angles and fractal dimension were statistically less affected by either exposure (Table 2).

**Table 2:** Summary statistics of the statistical tests estimating the significance of the effects of experimental conditions on the movement behaviour of *A*. *aquaticus*

	Resting times^a,b^		Step lengths^c,d^		Turning angle^e^			Fractal D^a,b,*^		Spearman’s rank correlation between turning angle and step length	
	*t*	*p*	U	*p*	W	*p*	*df*	*t*	*p*	r	*p*
*Pesticides*											
Chlorpyrifos low											
1	−3.26	<0.01	238	0.02	2.24	0.33	2	0.22	0.83	−0.29	<0.01
50	−0.08	0.94	246	<0.01	2.23	0.33	2	−2.20	0.03	−0.38	<0.01
Chlorpyrifos high											
1	−3.74	<0.01	312	<0.01	5.96	0.05	2	−1.73	0.09	−0.49	<0.01
50	−1.05	0.31	233	<0.01	4.37	0.11	2	−0.54	0.59	−0.40	<0.01
Imidacloprid low											
1	−3.10	<0.01	330	<0.01	6.70	0.04	2	1.01	0.32	−0.41	<0.01
50	−1.16	0.26	298	<0.01	0.37	0.83	2	−1.55	0.13	−0.42	<0.01
Imidacloprid high											
1	−2.25	0.03	340	<0.01	3.83	0.15	2	1.36	0.18	−0.51	<0.01
50	−0.75	0.46	247	0.05	3.89	0.14	2	1.97	0.06	−0.36	<0.01
*Controls*											
Control (starved)											
1	−2.43	0.02	226	0.19	4.78	0.09	2	1.93	0.06	−0.25	<0.01
50	−2.12	0.04	311	<0.01	3.89	0.14	2	−0.71	0.48	−0.23	<0.01
Control (fed)											
1										−0.25	<0.01
50										−0.39	<0.01
*Environmental factors*											
Food											
1	−1.19	0.32	235	0.35	3.73	0.15	2	0.65	0.52	−0.22	<0.01
50	−0.84	0.41	233	0.06	0.91	0.63	2	1.72	0.10	−0.21	<0.01
Shelter											
1	−0.87	0.39	217	0.46	5.25	0.07	2	1.05	0.30	−0.34	<0.01
50	−0.35	0.73	221	0.24	4.15	0.13	2	−0.90	0.38	−0.43	<0.01

	*df*	F	*p*	*df*	Χ^2^	*p*	*df*	W	*p*	*df*	F	*p*
*Pesticide concentrations*												
Chlorpyrifos												
1	28.4	10.75	<0.01	2	18.69	<0.01	4	7.42	0.12	35.75	2.15	0.13
50	35.9	5.71	<0.01	2	17.94	<0.01	4	12.92	0.01	36.97	0.94	0.40
Imidacloprid												
1	28.8	0.55	0.59	2	9.71	<0.01	4	4.57	0.33	36.35	2.73	0.08
50	33.5	0.75	0.48	2	9.23	0.01	4	3.90	0.42	37.16	6.67	<0.01

Parametric tests were applied for evaluating effects on resting times and a transformed version of the fractal dimension, while non-parametric tests were chosen in the case of step lengths and turning angles. For additional insights into effect sizes, the correlations of step lengths and turning angles were estimated for each treatment

^a^Welch’s *t* test for 2-sample comparison

^b^Welch’s ANOVA for multi-sample comparison

^c^Mann–Whitney U test for 2-sample comparison

^d^Kruskal–Wallis test for multi-sample comparison

^e^Watson–Wheeler test for 2- and multi-sample comparison

* Fractal dimension was log(D-1) transformed prior to statistical testing

#### Environmental stimuli

Observed R_n_^2^ were more similar to each other in the food, shelter, and their respective control tests (Fig. 2f–h) than was the case for the pesticide tests. The presence of food items slightly decreased R_n_^2^ in the single individual setup, whereas the presence of shelter items did not cause any observable changes. The biggest effect on observed R_n_^2^ in these three setups was caused by population density. Higher population densities led to decreased R_n_^2^ (Fig. 2f–h). Resting times increased compared to the controls when shelter or food items were introduced to the arena (Table 2). In the presence of shelter, resting times were equal among the different population densities. When food items were present, the single- and 50-individual specimen maintained the approximate 10 % difference that we also found in the control groups. Average step lengths remained virtually the same in the presence of food items, and were slightly lower, although not significant, when shelter items were available. Amongst the different treatments, the observed individuals increased resting times and decreased average step lengths when they were with conspecifics compared to the respective single-specimen setups, probably due to the increased “traffic”. Average turning angles increased in the presence of food items, while the presence of shelter items left this parameter unaffected. The fractal dimension decreased slightly more when shelter items were available than when food items were present (Table 1). The variability of these parameters was less affected by either treatment than observed in the pesticide exposure experiments, and no statistical indication of treatment effects could be detected. These changes indicate that the observed *Asellus* started searching for food when food items were present, while the presence of shelter provided structures for resting.

Food availability before the experiments had the overall biggest influence on the observed movement behaviour. The pesticide control groups did not receive food for 48 h prior to the experiment. The control groups for testing the influence of external factors, on the other hand, had access to food until shortly before the recording. The lack of food caused an increase in observed net-squared displacement (Fig. 2a, f), which can be explained by a statistically significant reduced resting time and increased step lengths (Table 1). While the turning angle range hardly changed, the fractal dimension decreased slightly, indicating that the observed animals changed to overall more linear movements. Additionally, the differences in resting times and step lengths found between the single- and 50-specimen setups disappeared when the individuals were starved (Table 1).

### Correlation and autocorrelation

Most observed individuals in the various treatments displayed directional persistence forwards (Table 2), meeting the central assumption made under the CRW framework. Turning angles were also significant positively auto-correlated at lag 1 in most cases, and remained significant for several lags (see Online Resource 2 for detailed results), representing a tendency to make sequential turns in the same direction. Furthermore, auto-correlations in step lengths were significant positive at lag 1 for almost all individuals, and remained significant for a number of lags (Online Resource 2), which suggests that most individuals maintained similar walking speeds for a number of steps. In all treatments, step lengths and turning angles were significant negatively correlated (Table 2), i.e. larger changes in direction were performed only when the individuals slowed down, and average angles decreased with increasing walking speed.

### Dispersal estimates

Figure 2, furthermore, compares the observed and estimated net-squared displacements (R_n_^2^) of *A*. *aquaticus* under different testing conditions. The CRW model overpredicts observed R_n_^2^ in cases where the observed path is more tortuous than assumed by the model. In cases of underestimation, the observed path is straighter or the animal activity lower than expected.

Generally, we found that estimated R_n_^2^ exceeded the observed values for the non-pesticide, single-specimen observations, while observed R_n_^2^ were mostly underestimated after pesticide exposure. Exceptions are the lower chlorpyrifos and the starved control treatments. At the higher population density this pattern changes and all observed R_n_^2^ exceed the estimated values except for the starved control group (Fig. 2a–e). In the latter case, the model fits the observed pattern better for the non-pesticide treatments during the initial steps compared to the pesticide treatments. However, the CRW models do not provide a good overall fit to the observed displacements (Fig. 2). The closest fits were found for the higher population density when the observed individuals were fed, and when food items were present (Fig. 2g).

## Discussion

This study aimed to improve insights into the small-scale movement behaviour of *A*. *aquaticus* and to evaluate its potential as endpoint in ecotoxicological studies with aquatic macroinvertebrates. The employed video-tracking method (Augusiak and Van den Brink 2015) allowed the detection of already small changes in the exhibited behaviour, although the high inter-individual variability of the analysed parameters made it difficult to detect statistical significant treatment effects. Our results indicate that the locomotory behaviour and dispersal potential of *A*. *aquaticus* were negatively affected by exposure to sublethal concentrations of chlorpyrifos and imidacloprid, while the presence of food or shelter items reduced the dispersal rate less significantly. In most cases, an increased population density lowered dispersal rates further. The observed effects on the small-scale behaviour also affected the displacement extrapolations.

The pesticides were chosen because of their relatively low elimination rates, making it likely that exposed individuals still experience pesticide related effects when placed in clean water that then can be observed. Rubach et al. (2010) report a 95 % depuration time of 16.2 days for chlorpyrifos in *A*. *aquaticus* and of 7.5 days for adult *Gammarus pulex*, a freshwater shrimp species. In the case of imidacloprid, Ashauer et al. (2010) determined a 95 % depuration period of 11.2 days for *G*. *pulex*. We assumed a continued causation of damage on the nervous system of *A*. *aquaticus* during the experimental time frame also in the case of imidacloprid. First estimations based on acute toxicity data of imidacloprid exposure, yielded a 95 % depuration period of about 4.4 days for *Asellus* (Focks 2015—personal communication).

The fact that *G*. *pulex* exhibits significantly higher sensitivities to both chemicals with regard to mobility and survival indicates that surviving individuals could possess a more efficient elimination pathway compared to *Asellus*, allowing the conclusion that the internal concentrations in our study should be stable over the period of time of observation. To test whether changes in locomotion are still observable at sublethal levels, we aimed to apply about 50 and 25 %, respectively, of the observed 48 h-EC10 of 2.7 μg/L in the case of chlorpyrifos (Rubach et al. 2011: 48 h-EC10 = 3.3 μg/L). Due to a wider range of reported ECx values, we opted for a slightly higher safety factor for imidacloprid and chose to continue with about 30 and 15 %, respectively, of the observed 48 h-EC10 value of 225 μg/L (geometric mean of studies reported by Roessink et al. (2013) and Van den Brink et al. (2015): 48 h-EC10 = 54 μg/L). The applied concentrations are also likely to occur in the environment. Concentrations of up to 10.8 μg/L of chlorpyrifos were detected in freshwater habitats throughout the past decade (Marino and Ronco 2005; Ensminger et al. 2013), while imidacloprid has been found at concentrations of up to 320 μg/L (Van Dijk et al. 2013; Ensminger et al. 2013).

In natural environments, the dispersal and local recruitment of aquatic macroinvertebrates is strongly driven by the availability of food, shelter, and population density (Holyoak et al. 2008). Food items may release chemicals during the degradation process, which then can be sensed by an organism equipped with the respective sensing systems (Collin and Marshall 2003). This can subsequently cause an alteration in the organism’s searching behaviour, for example a switch from long, straight moves to a Brownian pattern for local searching together with a change of activity (Collin and Marshall 2003). Similarly, a lack of food may drive animals away from their current location to search for new resources. Shelter, on the other hand, can impact overall movement by providing protection from high temperatures, light, or predators (Obermüller et al. 2007). However, there is a lack of understanding to which degree the presence of food or shelter items can influence the movement and searching behaviour of aquatic invertebrates, or how it may additionally be driven by population density, either by compensating for interspecies competition or improving mating chances (Smith et al. 2008; Delgado et al. 2013).

Understanding the innate nature of movement behaviour, and to which degree different factors influence it, can help extrapolating small-scale observations to gain an impression on the ecological consequences of chemical or physical disturbances (Getz and Saltz 2008). In Table 3, we summarize a number of studies aiming to highlight the influences of chemical exposure or naturally occurring drivers, such as predator cues, on the movement behaviour of aquatic macro invertebrates. We found that most published studies on aquatic invertebrates either focused on environmental cues or chemical exposure, while none related the extent of behavioural changes under sublethal exposure conditions to the innate behavioural range to draw conclusions about potential ecological impact. Observational studies that do investigate such relationships usually use food consumption rates or preferences as endpoint instead of movement (for examples see De Lange et al. 2006b; Agatz et al. 2014). The study by (Rodrigues et al. 2016) forms a rare exception, where the effects of sublethal exposure of freshwater planarians to chlorantraniliprole are investigated through observing changes in feeding behaviour and locomotion.

**Table 3:** Literature survey of studies investigating the influence of chemicals and/or environmental conditions on aquatic macroinvertebrate locomotion in the laboratory

Observational method	Species	Experimental dimension	Variable	Movement related metrics	Reference
Camera	*A*. *aquaticus*, *Gammarus pulex*	Aquaria (100 L)	Population density	Speed, turning angles, fractal dimension	Augusiak and Van den Brink (2015)
	*Acilius sulcatus*	Aquaria (100 L)	Kairomones	Distance	Åbjörnsson et al. (1997)
	*Balanus amphitrite*	Petri dishes	Various antifouling biocides, Heavy metals, Neurotoxic pesticides	Swimming speed	Faimali et al. (2006)
	*Brachionus calyciflorus*	Glass chamber	Copper, Pentachlorophenol (PCP), Lindane	Speed, sinuosity	Charoy and Janssen (1999)
			Food presence, nutritive state		Charoy (1995)
			Copper, Pentachlorophenol (PCP), Lindane, 3,4-dichloroaniline		Charoy et al. (1995)
		Well-plates	Dimethoate	Speed, sinuosity, turning angles	Guo et al. (2012)
	*Brachionus calyciflorus*, *Asplanchna brightwelli*	Well-plates	Dimethoate	Speed	Chen et al. (2014)
	*Brachionus plicatilis*, *Artemia sp*.	Petri dishes, well-plates	Zinc pyrithione, Macrotrol^®^ mt-200, Eserine	Speed	Garaventa et al. (2010)
	*Daphnia pulex*	Exposure cells (20 mL)	Isopropanol, Ethanol, Caffeine, Imidacloprid, Sertraline, Copper sulfate, Fipronil, Carbofuran, Esfenvalerate, Cypermethrin, Abamectin, Trichlorfon	Speed, turning angles, activity	Chevalier et al. (2015)
		Beaker (200 mL)	Carbaryl, Kairomones	Speed, turning angles, diel movement	Dodson et al. (1995)
		Well-plates	Chlorpyrifos, Nicotine, Physostigmine	Distance, turning angles	Zein et al. (2014)
	*Eurytemora affinis*	Beaker (200 mL)	Nonylphenols	Speed	Cailleaud et al. (2011)
	*Gammarus pulex*	Petri dishes, stream mesocosms	Lambda-cyhalothrin	Speed, activity, drift	Nørum et al. (2010)
		Petri dishes	Cypermethrin	Speed, activity	Nørum et al. (2011)
	*Litopenaeus vannamei*	Aquaria (7 L)	Methamidophos	Activity, qual. Observations	García-de la Parra et al. (2006)
	*Oncaea venusta*	Small plastic tanks	Inherent individual variability	Speed, distance	Seuront et al. (2004a, b)
	*Rana temporaria tadpoles*	Small plastic tanks	Endosulfan	Speed, activity	Denoël et al. (2013)
Multispecies freshwater biomonitor	*Chironomus larvae*	Beaker (ca 200 mL)	Imidacloprid	Ventilation, activity	Azevedo-Pereira et al. (2011)
	*Daphnia magna*		Dipterex, Malathion, Parathion, Dimethyl sulfoxide	Motility	Ren et al. (2007)
			Dichlorvos, Malathion, Parathion, Methyl parathion		Ren et al. (2008)
	*Gammarus pulex*		Pharmaceuticals	Ventilation, activity	De Lange et al. (2006a, 2009)
			Time of day	Ventilation, activity	Peeters et al. (2009)
	*Echinogammarus meridionalis*, *Hydropsyche pellucidula*, *Choroterpes picteti*		Acidic mine drainage	Ventilation, activity	Macedo-Sousa et al. (2008)
Visual inspection	*A*. *aquaticus*, *Dendrocoelum lacteum*	Crystallization dishes (500 mL)	Tebuconazole, Lambda-cyhalothrin	Activity, predator–prey interaction	Bundschuh et al. (2012)
	*A*. *aquaticus*, *Gammarus pulex*	Aquaria (1.5 L)	Polycyclic aromatic hydrocarbons	Avoidance	De Lange et al. (2006b)
	*Brachionus calyciflorus*	Glass chamber	Copper, Pentachlorophenol (PCP), Lindane, 3,4-dichloroaniline	Distance walked	Janssen et al. (1994)
	*Chaoborus flavicans larvae*	Aquaria (12 L)	Kairomones	Height in water column	Dawidowicz et al. (1990)
	*Rana catesbeiana tadpoles*, *Rana septentrionalis tadpoles*	Aquaria (15 L)	Kairomones	Mobility	Ferland-Raymond et al. (2010)

The strong reductions in observed dispersal distances after pesticide exposure were mostly caused by decreased step lengths and increased resting times, which agrees with previous reports of hypoactivity caused by both substances (Rice et al. 1997; Suchail et al. 2001). Step lengths were significantly reduced by all pesticide treatments, while resting time was more affected by exposure to chlorpyrifos than to imidacloprid. The turning behaviour, i.e. directionality, was not significantly different from that observed in the controls after pesticide exposure, although the variability was higher after exposure (Table 2). These effects are in accordance with the modes of action of the used insecticides. Both substances disturb neural signal regulation to a degree that neurological activity of nerves remains lastingly stimulated, which eventually leads to muscle spasms and paralysis. Chlorpyrifos does so by inactivating the enzyme that hydrolyses acetylcholine, and imidacloprid by activating nACh receptor. The more pronounced effects we found in the case of chlorpyrifos exposure, i.e. the increase in resting time coupled with a decrease in average step length, might be associated with the irreversibility of the enzyme activation, while the nAChR stimulation through imidacloprid is reversible. The reduced step lengths and changes in resting behaviour indicate that muscle malfunction may have set in already at the time of observation. The increased variability of turning angles can be explained by either muscular impairment or additional neurological effects affecting the individuals’ ability to navigate. Based on a study by Azevedo-Pereira et al. (2011) we would speculate to find effects of exposure to chlorpyrifos and imidacloprid to converge further after an extended exposure duration or at increased concentrations. In their study, Azevedo-Pereira et al. (2011) measured AChE activity along with behavioural endpoints after exposure of *Chironomus riparius* larvae to imidacloprid and found that AChE activity also decreased with increasing concentration after 96 h of exposure onward. The chain of physiological effects of AChE inhibition in *Asellus*, respectively, would lead to a decrease in overall activity as would be the case after exposure to chlorpyrifos, which directly inhibits AChE activity.

Dose–response or population density related effects were less conclusive in our study. While at the higher concentrations, the higher population densities appear to incite higher activity and slightly larger step lengths, compared to their single-individual equivalents, no such pattern could be identified for the lower concentration treatments. This aspect, together with the high individual variability in behaviour only demonstrates that more research is needed fully understand the sublethal impacts of pesticide exposure on ecologically relevant functions. Eventually, reduced locomotion is likely to interfere with foraging activities as observed by Agatz et al. (2014) in the case of Gammarids. Decreased energy available from feeding and increased energy expenditure for internal repair mechanisms, in turn, may lead to reduced growth and mating (Martin et al. 2012).

In our study, the impact on organisms exposed to imidacloprid may be less drastic compared to chlorpyrifos due to the higher safety factor that we assumed. However, the significance of pesticide exposure becomes clearer, when seen in comparison to the non-pesticide treatments. The presence of food slightly lowered the dispersal potential by affecting orientation moments and variation of turning angles, indicating that the animals were indeed adjusting their searching efficiency. Shelter items on the other hand caused a comparable reduction in dispersal. However, mechanistically it resulted from an effect on activity by reducing step lengths and increasing resting times. The presence of conspecifics affected reorientation less as could probably be expected than that it increased resting times in most cases, respectively reducing overall dispersal. The differences between the fed and starved control groups, however, indicate that the feeding state could potentially change this and reduce the need of shelter availability.

To improve the risk level estimation of chemical exposure on aquatic arthropod populations in an ecologically more meaningful way, ecological effect models can be applied that integrate different habitat, species, and exposure related information to assess population recovery timeframes (Galic et al. 2013; Focks et al. 2014). Accounting for immigrating and emigrating individuals can help to further the mechanistic understanding derived from such modelling studies (Van den Brink et al. 2013; Hommen et al. 2015). The simplified dispersal estimation via the correlated random walk framework as part of this study failed to capture the underlying correlations between turning angles and step lengths, as well as the autocorrelation structures of either of these two parameters. Westerberg et al. 2008 studied the effects of population density and food availability on collembola described a similar phenomenon. The mechanistic links of the *Asellus* decision making remain to be elaborated for a better model parameterization. Aggregating the step length data may be one of those approaches to eliminate the CRW assumption of non-autocorrelated steps. The high variability of individual behaviour expressions is another factor that complicates simple modelling approaches, although it is an often observed factor in observational studies (Seuront et al. 2004a; Nørum et al. 2010). Hawkes (2009) consequently propose to account explicitly for this variability when designing models of habitat use and dispersal, respectively, an approach that is ignored by the application of simple average values in our study. Integrating findings such as ours into a more complex model can facilitate a better understanding of the complex interactions of chemical exposure and resource availability and their impacts on population recovery times, allowing also for the study of long-term impacts of exposure events.

## Electronic supplementary material

Below is the link to the electronic supplementary material.

Online Resource 1: Results of a 48 h toxicity test for determining exposure concentrations of imidacloprid and chlorpyrifos in this study. (PDF 42 kb)

Online Resource 2: Detailed results of the movement behaviour study. (PDF 10326 kb)
