# Supplementary material for: The influence of insecticide exposure and environmental stimuli on the movement behaviour and dispersal of a freshwater isopod
Source: Ecotoxicology. 2016 Jun 15;25:1338–52. doi: 10.1007/s10646-016-1686-y (PMC4961728; doi:10.1007/s10646-016-1686-y)
Supplement: Supplementary file 1 — Online Resource 1: Results of a 48 h toxicity test for determining exposure concentrations of imidacloprid and chlorpyrifos in this study. (PDF 42 kb) [file 10646_2016_1686_MOESM1_ESM.pdf]

Online Resource 1:

The influence of insecticide exposure and environmental stimuli on the movement behaviour and dispersal of a freshwater isopod

Jacqueline Augusiak[1], Paul J. Van den Brink[1,2]

- Ecotoxicology -

[1] Wageningen University, Aquatic Ecology and Water Quality Management Group, Wageningen University and Research centre, P.O. Box 47, 6700 AA Wageningen, The Netherlands

[2] Alterra, Wageningen University and Research centre, P.O. Box 47, 6700 AA Wageningen, The Netherlands

Corresponding author

Name: Jacqueline Augusiak

Phone: +31 317 48 59 58

Fax: +31 317 41 90 00

Email: jacqueline.augusiak@wur.nl

Toxicity tests

To study the effects of the pesticides on mortality and immobilization, separate toxicity tests were conducted for each pesticide. Ideally, the concentrations chosen for the movement studies should be below the observed EC10 levels and lead to observable behavioural changes but not complete paralysis or death.

Additionally, the movement study was designed to make use of individuals that were exposed for 48 hours, then removed from the exposure solution, rinsed with copper free water and placed in cosms that contained clean copper free water. Thus, the studied substances should not be readily detoxified by the test species. Toxico-kinetic data were used as measure for depuration time (imidacloprid: Ashauer et al. (2010) and chlorpyrifos: Rubach et al. (2010)).

Materials & Methods

Exposure solutions of chlorpyrifos and imidacloprid were prepared as described in the main manuscript. For chlorpyrifos the selected exposure concentrations were 0, 0.6, 1.5, and 3 μg/L, for imidacloprid 0, 100, 200, 400, 800, 1600 μg/L. Three replicates with 10 animals each were prepared per concentration level and stainless steel hook-shaped gauze pieces were inserted into the beakers to provide a physical substrate for the animals. In the case of chlorpyrifos, 2.5L Weck beakers were used and filled with 1.5 L of exposure solution, in case of imidacloprid 1.5 L Weck beakers filled with 0.75 L exposure solution were used. Test animals ranged from 0.5 to 0.9 cm in body length and no artificial aeration was supplied to minimize the evaporation of the chemicals. Beakers were closed with lids throughout the test. The light/dark regime was adjusted to 16/8 hours. Water temperature, pH and dissolved oxygen were monitored in the beginning, and after 24 and 48 hours to confirm stable conditions throughout the experimental period. All experiments were carried out at a water temperature of 19.6 ± 1.0 ºC, an average pH of 8.0 ± 0.1 (measured with electrode pH323, WTW Germany) and an average dissolved oxygen level of 8.6 ± 0.7 mg/L (measured with oximeter Oxi330 equipped with sensor CellOx 325, WTW Germany), all corresponding to the levels occurring in the movement experiments.

To determine the chlorpyrifos concentrations, water samples from all test vessels were taken and analysed for chemical concentrations as described in the main document at t = 0 and after 24 and 48 hours. The test lasted 48 hours and animals were scored after 24 and 48 hours with regard to mobility and mortality. Criteria for defining these parameters were taken from Rubach et al. (2011).

The imidacloprid toxicity study lasted for 96 hours since no reported EC10 values for *A. aquaticus* could be found in the literature at the time of the study and values determined for other species covered a broad range. The numbers of immobile and dead animals were counted after 24, 48, 72, and 96 hours in each replicate. Water samples were taken from each beaker at the start of the experiment and after 96 hours, and processed as described in the main manuscript. However, samples with concentrations above 400 µg/L were diluted by factor 10 prior to analysis.

Results

Exposure

The measured exposure concentrations of chlorpyrifos and imidacloprid are given in Table S1.1. The chlorpyrifos concentrations in the exposure solutions were well within the range of the intended concentrations and decreased slightly over time as expected. Due to the high volatility of the substance and associated contamination routes, one of the controls received cross-contamination. This was tolerated because the control immobility and mortality were below 10%. Similarly, the nominal concentrations of imidacloprid were well achieved in the exposure solutions, which, remained more stable over time than was observed with chlorpyrifos. The increased variability of measured concentrations in the samples of the highest three concentration levels may be due to the dilution step.

Effects on mortality and mobility

For both pesticides, mortality and immobility increased with increasing concentrations, and over time (Table S1.1). This was more pronounced for imidacloprid than for chlorpyrifos in our study but was also expected for the chosen concentration ranges. Van Wijngaarden et al. (1996) report an 48h-EC10 for chlorpyrifos of 2 µg/L, and an 48h-EC50 of 4.3 µg/L, respectively, for *A. aquaticus* in laboratory based experiments. Rubach et al. (2011) found an 48h-EC50 of 6.16 µg/L (48h-EC10 = 3.3 µg/L) for*A. aquaticus* under similar conditions. Our results (see Table S1.2) fall into a comparable range.

Reported ECx and LCx values for imidacloprid exposure of *A. aquaticus* range wider than those for chlorpyrifos. Lukančič et al. (2010) determined a 48h-LC50 of 8.5 mg/L, and a 24h-EC50 of 0.8 mg/L for *A. aquaticus*. Roessink et al. (2013) found a 96h-LC50 of 316 µg/L (96h-LC10: 61.6 µg/L) and a 96h-EC50 of 119 µg/L (96h-EC10: 24.7 µg/L). Van den Brink et al. (2015) compared the work of Roessink et al. (2013) on a summer generation with an overwintering generation of Asellus and reported an additional 96h-EC50 of 78 µg/L. Similar

to chlorpyrifos, the results obtained for imidacloprid in this study (see Table S1.2) lie in between those reported levels.

Conclusions

Our findings correlate well with other studies, indicating that the population we worked with was similarly sensitive and, thus, representative. Furthermore, based on the findings and the motivation for conducting the toxicity experiments, we chose to continue the study of effects of sublethal exposure on the movement behaviour with the concentrations listed in Table S1.3.

Because we intended to investigate if concentrations below the immobilization level would already lead to observable changes in behaviour, we decided to continue working with about 50% and 25%, respectively, of the observed EC10 in the case of chlorpyrifos. We had more experience working with this substance in combination with the chosen model species. Respectively, we opted for a slightly higher safety factor for imidacloprid and chose to continue with about 30% and 15%, respectively, of the observed EC10 value. Both decisions were also driven by the fact that the resulting exposure concentrations are likely to occur in the field (Muschal and Warne 2003; Marino and Ronco 2005; Van Dijk et al. 2013; Ensminger et al. 2013; Agatz et al. 2014; Papadakis et al. 2015).

References

Agatz A, Ashauer R, Brown CD (2014) Imidacloprid perturbs feeding of Gammarus pulex at environmentally relevant concentrations. Environ Toxicol Chem 33:648–653. doi: 10.1002/etc.2480

Ashauer R, Caravatti I, Hintermeister A, Escher BI (2010) Bioaccumulation kinetics of organic xenobiotic pollutants in the freshwater invertebrate Gammarus pulex modeled with prediction intervals. Environ Toxicol Chem 29:1625–1636. doi: 10.1002/etc.175

Ensminger MP, Budd R, Kelley KC, Goh KS (2013) Pesticide occurrence and aquatic benchmark exceedances in urban surface waters and sediments in three urban areas of California, USA, 2008-2011. Environ Monit Assess 185:3697–3710. doi: 10.1007/s10661-012-2821-8

Lukančič S, Žibrat U, Mezek T, et al (2010) A new method for early assessment of effects of exposing two non-target crustacean species, Asellus aquaticus and Gammarus fossarum, to pesticides, a laboratory study. Toxicol Ind Health 26:217–228. doi: 10.1177/0748233710362379

Marino D, Ronco A (2005) Cypermethrin and Chlorpyrifos Concentration Levels in Surface Water Bodies of the Pampa Ondulada, Argentina. Bull Environ Contam Toxicol 75:820–826. doi: 10.1007/s00128-005-0824-7

Muschal M, Warne MSJ (2003) Risk Posed by Pesticides to Aquatic Organisms in Rivers of Northern Inland New South Wales, Australia. Hum Ecol Risk Assess An Int J 9:1765–1787. doi: 10.1080/714044796

Papadakis E-N, Tsaboula A, Kotopoulou A, et al (2015) Pesticides in the surface waters of Lake Vistonis Basin, Greece: Occurrence and environmental risk assessment. Sci Total Environ 536:793–802. doi: 10.1016/j.scitotenv.2015.07.099

Roessink I, Merga LB, Zweers HJ, Van den Brink PJ (2013) The neonicotinoid imidacloprid shows high chronic toxicity to mayfly nymphs. Environ Toxicol Chem 32:1096–1100. doi: 10.1002/etc.2201

Rubach MN, Ashauer R, Maund SJ, et al (2010) Toxicokinetic variation in 15 freshwater arthropod species exposed to the insecticide chlorpyrifos. Environ Toxicol Chem 29:2225–2234. doi: 10.1002/etc.273

Rubach MN, Crum SJH, Van den Brink PJ (2011) Variability in the dynamics of mortality and immobility responses of freshwater arthropods exposed to chlorpyrifos. Arch Environ Contam Toxicol 60:708–721. doi: 10.1007/s00244-010-9582-6

Van den Brink PJ, Van Smeden JM, Bekele RS, et al (2015) Acute and chronic toxicity of neonicotinoids to nymphs of a mayfly species and some notes on seasonal differences. Environ Toxicol Chem. doi: 10.1002/etc.3152

Van Dijk TC, Van Staalduinen MA, Van der Sluijs JP (2013) Macro-invertebrate decline in surface water polluted with imidacloprid. PLoS One 8:e62374. doi: 10.1371/journal.pone.0062374

Van Wijngaarden RPA, Van den Brink PJ, Crum SJH, et al (1996) Effects of the insecticide Dursban® 4E (active ingredient chlorpyrifos) in outdoor experimental ditches: I. Comparison of short-term toxicity between the laboratory and the field. Environ Toxicol Chem 15:1133–1142.

Table S1.1: Test concentrations of chlorpyrifos and imidacloprid over, including controls, and results of the acute toxicity studies given as percentage of affected individuals.

		**Immobility**				**Mortality**				**Measured concentration [µg/L] (± SD)**			
		24 h	48 h	72 h	96 h	24 h	48 h	72 h	96 h	0 h	24 h	48 h	96 h
Chlorpyrifos													
	**Control**	0%	3%	-	-	0%	3%	-	-	0 (± 0.00)	0 (± 0.00)	0.02 (± 0.01)	-
	**0.6 µg/L**	3%	7%	-	-	3%	7%	-	-	0.62 (± 0.00)	0.54 (± 0.04)	0.45 (± 0.01)	-
	**1.5 µg/L**	0%	3%	-	-	0%	3%	-	-	1.26 (± 0.00)	1.14 (± 0.02)	1.09 (± 0.02)	-
	**3.0 µg/L**	17%	40%	-	-	10%	23%	-	-	3.04 (± 0.00)	2.5 (± 0.07)	2.24 (± 0.01)	-
Imidacloprid													
	**Control**	0%	0%	0%	0%	0%	0%	0%	0%	0 (± 0.00)	-	-	0.3 (± 0.05)
	**100 µg/L**	0%	0%	3%	3%	0%	0%	0%	0%	97.47 (± 0.53)	-	-	97.64 (± 1.44)
	**200 µg/L**	3%	10%	10%	17%	3%	3%	3%	3%	164.99 (± 11.63)	-	-	162.38 (± 5.85)
	**400 µg/L**	20%	30%	37%	37%	7%	10%	20%	23%	381.25 (± 42.59)	-	-	429.54 (± 2.56)
	**800 µg/L**	60%	67%	67%	83%	33%	47%	57%	67%	778.8 (± 62.27)	-	-	829.16 (± 23.10)
	**1600 µg/L**	83%	90%	90%	97%	23%	40%	53%	70%	1651.75 (± 194.90)	-	-	1544.5 (± 7.30)

Table S1.2: Results of the acute toxicity study summarised as concentrations causing 10% or 50% lethality or immobility (LC10 and LC50, or EC10 and EC50, respectively; plus 95% confidence intervals) after 48 hours.

	**Mortality**				**Immobilization**			
	LC50 (µg/L)	(95% CI)	LC10 (µg/L)	(95% CI)	EC50 (µg/L)	(95% CI)	EC10 (µg/L)	(95% CI)
Chlorpyrifos	3.4	NC	2.8	NC	3.2	NC	2.7	NC
Imidacloprid	1517	(989-2327)	332	(191-578)	603	(487-747)	225	(155-326)

NC: could not be calculated

Table S1.3: Concentrations of chlorpyrifos and imidacloprid selected for studying the effects of acute sublethal exposure on the movement behaviour of *A. aquaticus.*

Chlorpyrifos		
	**Control**	0 µg/L
	**Low**	0.6 µg/L
	**High**	1.5 µg/L
Imidacloprid		
	**Control**	0 µg/L
	**Low**	37.5 µg/L
	**High**	75.0 µg/L
