# Supplementary material for: The influence of insecticide exposure and environmental stimuli on the movement behaviour and dispersal of a freshwater isopod
Source: Ecotoxicology. 2016 Jun 15;25:1338–52. doi: 10.1007/s10646-016-1686-y (PMC4961728; doi:10.1007/s10646-016-1686-y)

Online Resource 2:

The influence of insecticide exposure and environmental stimuli on the movement behaviour and dispersal of a freshwater isopod

Jacqueline Augusiak[1], Paul J. Van den Brink[1,2]

- Ecotoxicology -

[1] Wageningen University, Aquatic Ecology and Water Quality Management Group, Wageningen University and Research centre, P.O. Box 47, 6700 AA Wageningen, The Netherlands

[2] Alterra, Wageningen University and Research centre, P.O. Box 47, 6700 AA Wageningen, The Netherlands

Corresponding author

Name: Jacqueline Augusiak

Phone: +31 317 48 59 58

Fax: +31 317 41 90 00

Email: jacqueline.augusiak@wur.nl

Figure S2.1: Resting time distributions exhibited by observed *Asellus aquaticus* under different experimental conditions.

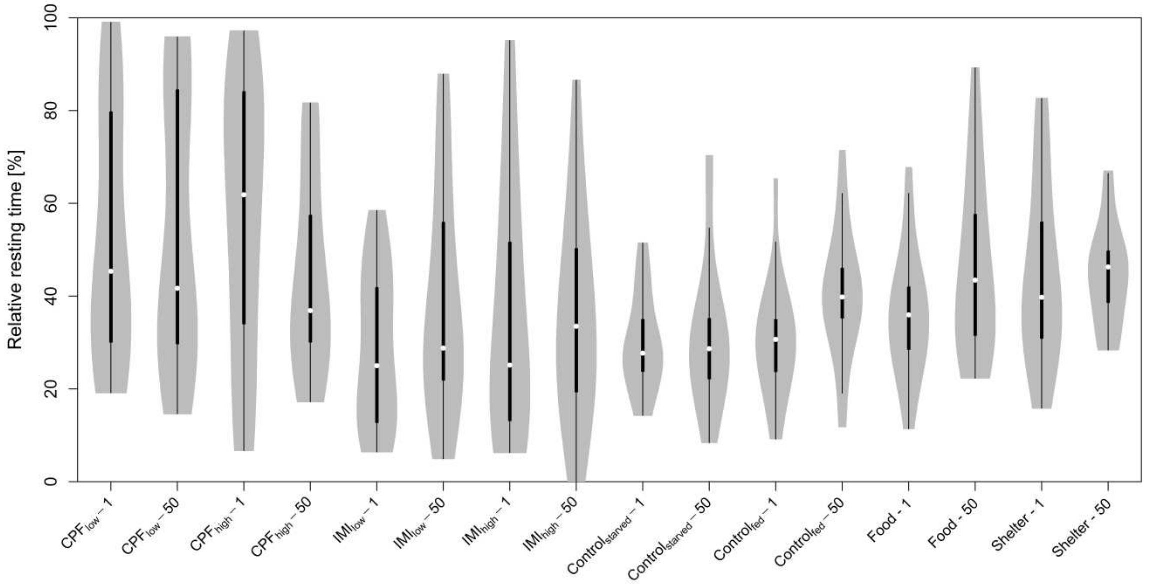

Figure S2.2: Step length distributions exhibited by observed *Asellus aquaticus* under different experimental conditions.

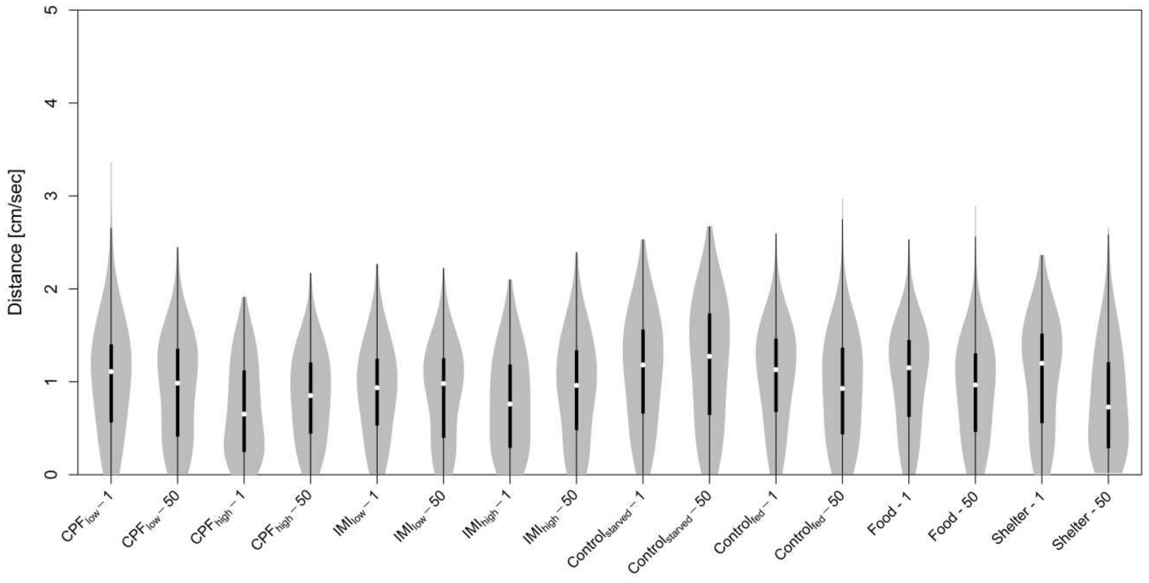

Figure S2.3: Turning angle distributions exhibited by observed *Asellus aquaticus* under different experimental conditions.

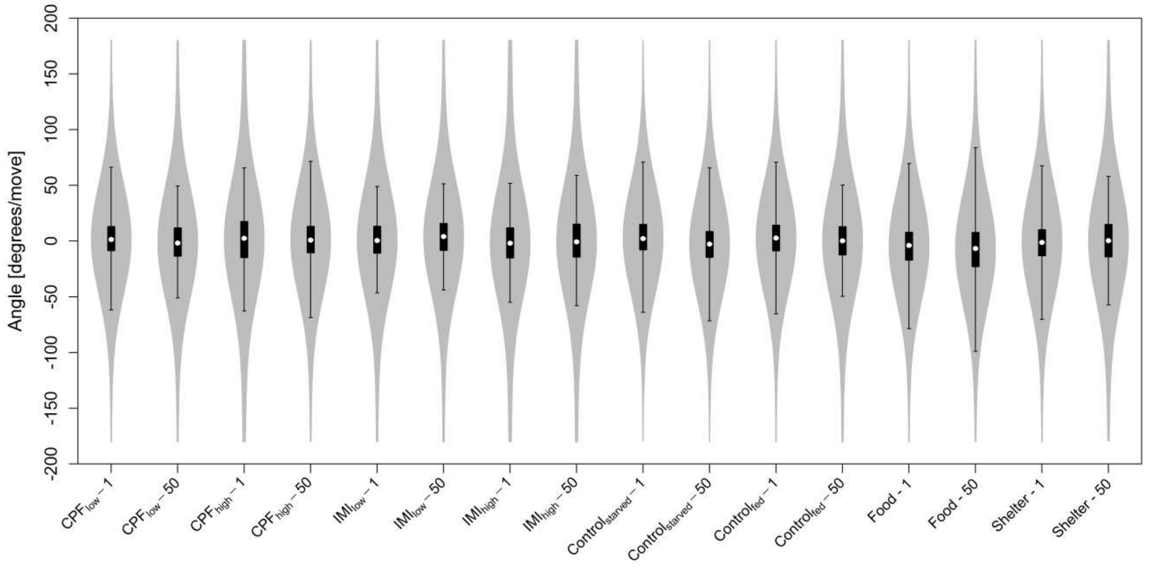

Figure S2.4: Distributions of the fractal dimension D exhibited by observed *Asellus aquaticus* under different experimental conditions.

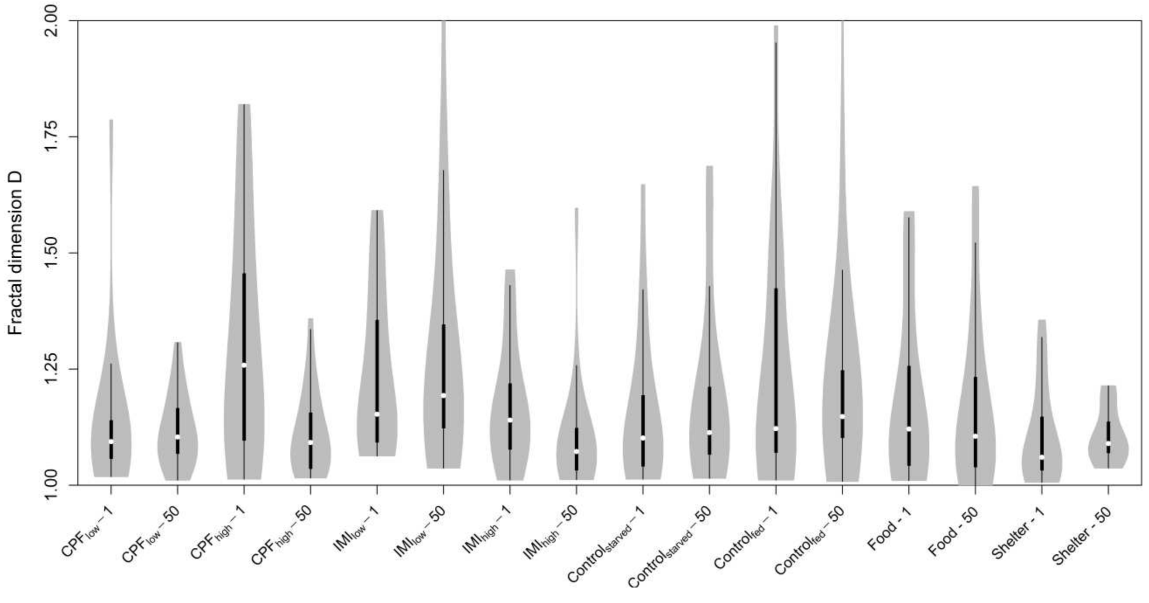

Figure S2.5: Correlations between the absolute turning angles (focussing on the turning sharpness rather than left or right orientation) and step lengths exhibited by observed *Asellus aquaticus* under different experimental conditions.

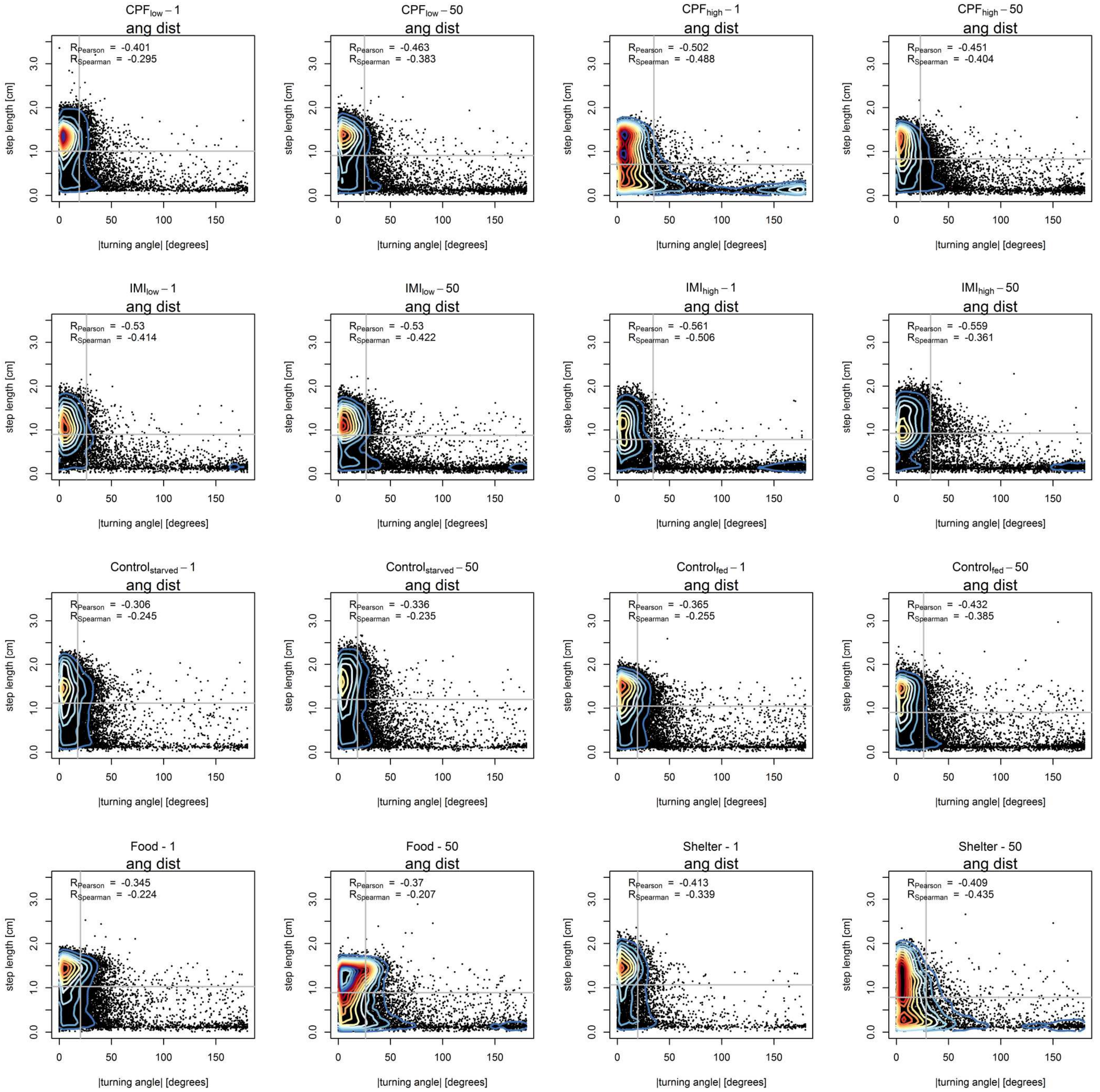

Figure S2.6a: Autocorrelation of step lengths exhibited by each observed individual over 10 lags.

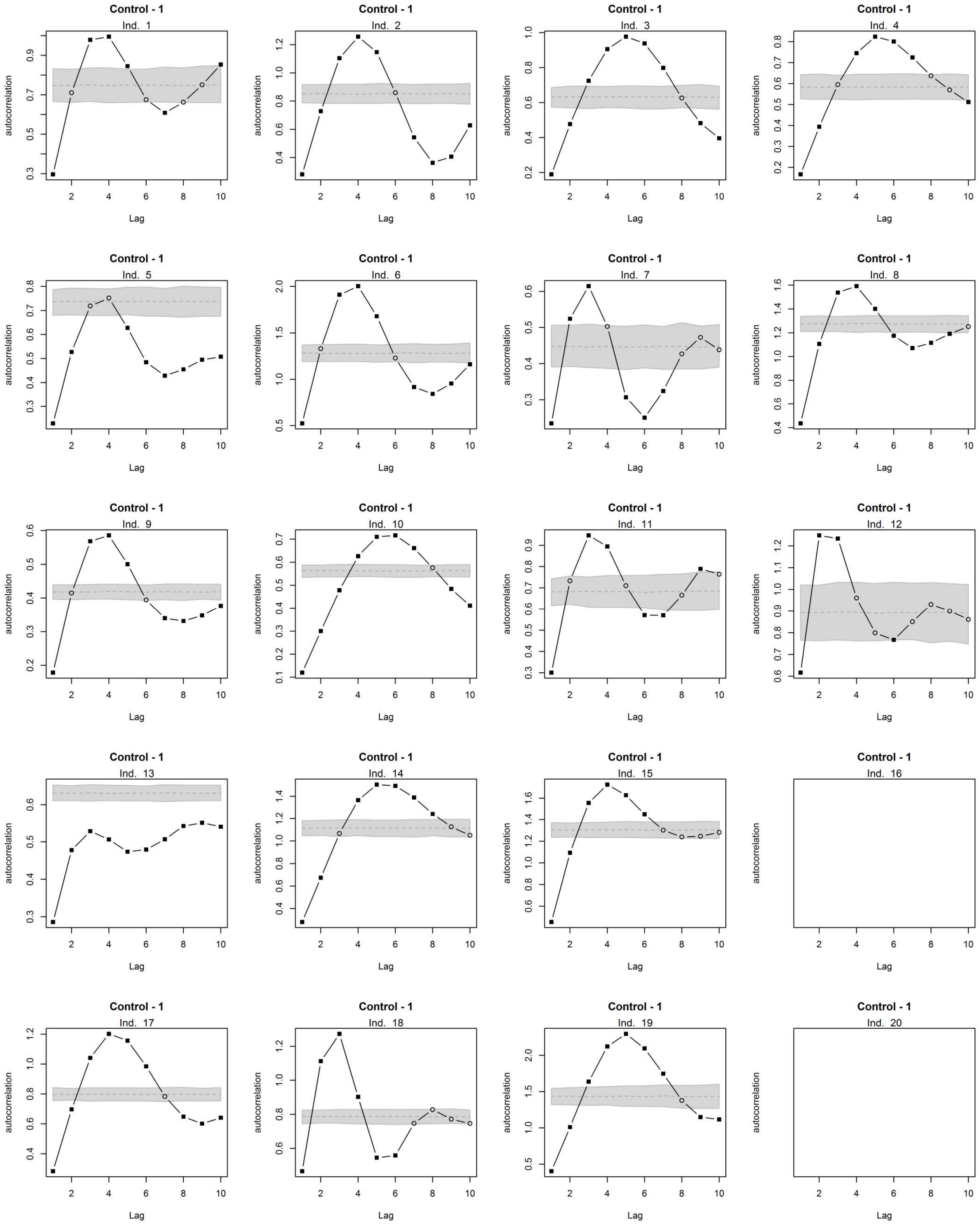

Figure S2.6b: Autocorrelation of step lengths exhibited by each observed individual over 10 lags.

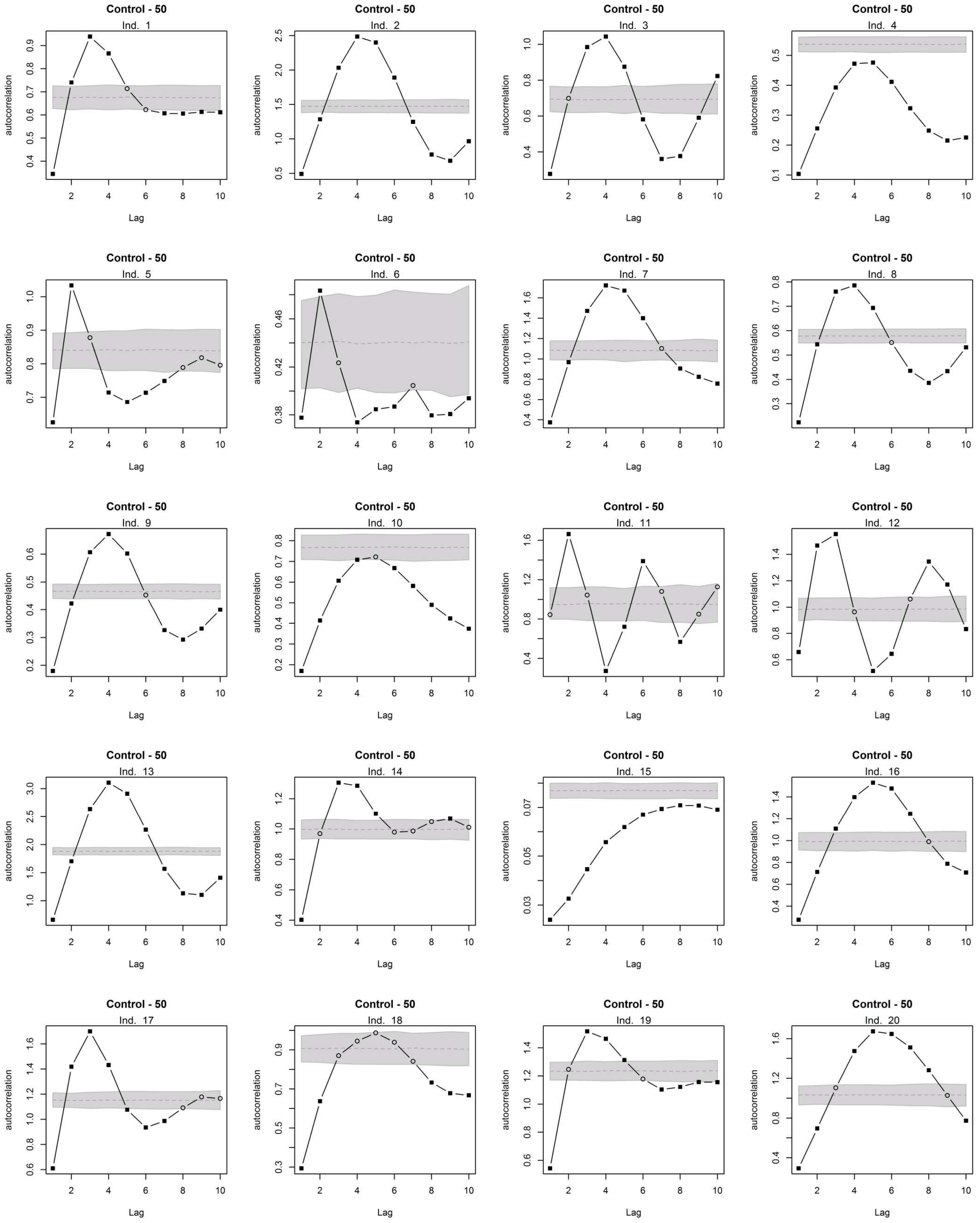

Figure S2.6c: Autocorrelation of step lengths exhibited by each observed individual over 10 lags.

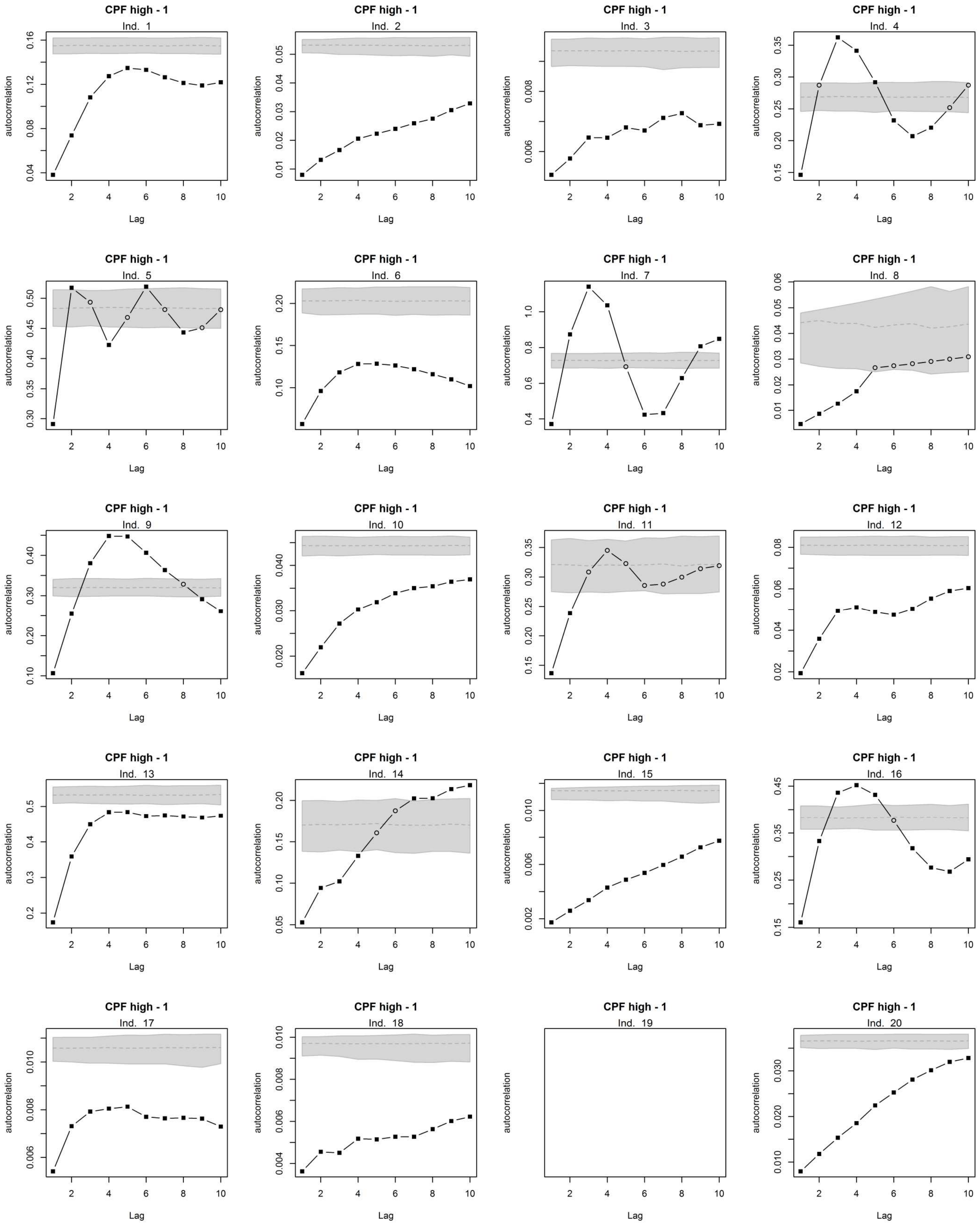

Figure S2.6d: Autocorrelation of step lengths exhibited by each observed individual over 10 lags.

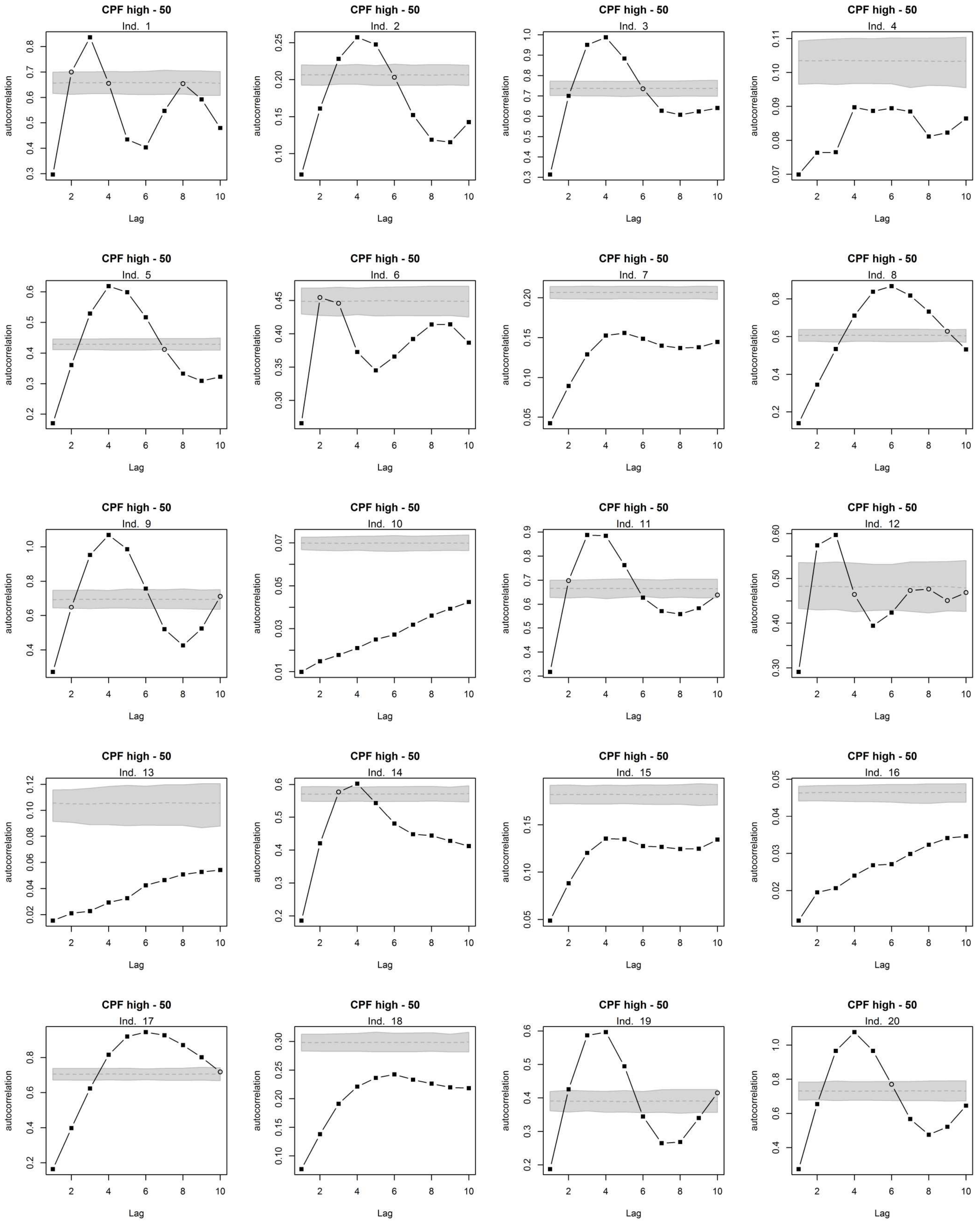

Figure S2.6e: Autocorrelation of step lengths exhibited by each observed individual over 10 lags.

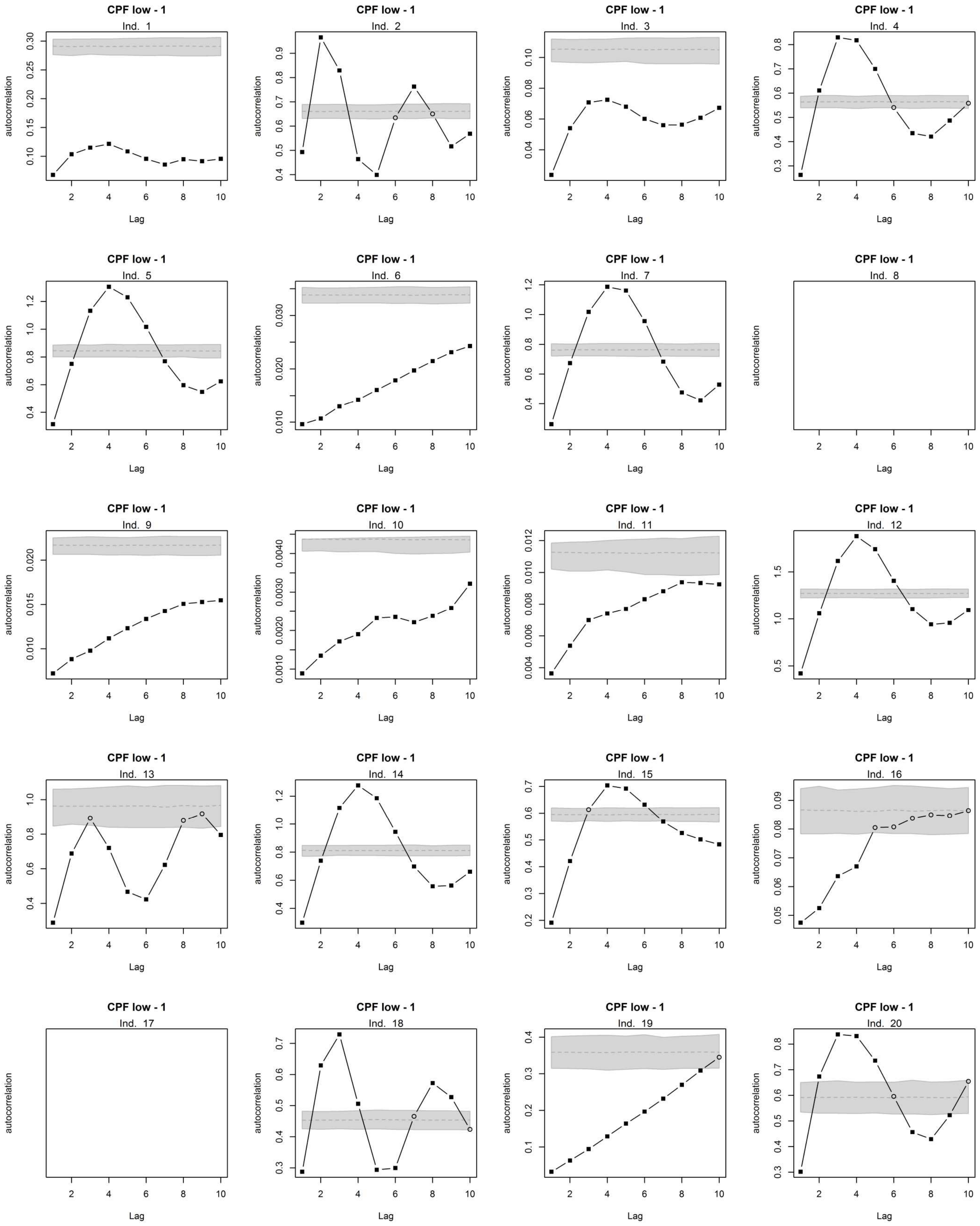

Figure S2.6f: Autocorrelation of step lengths exhibited by each observed individual over 10 lags.

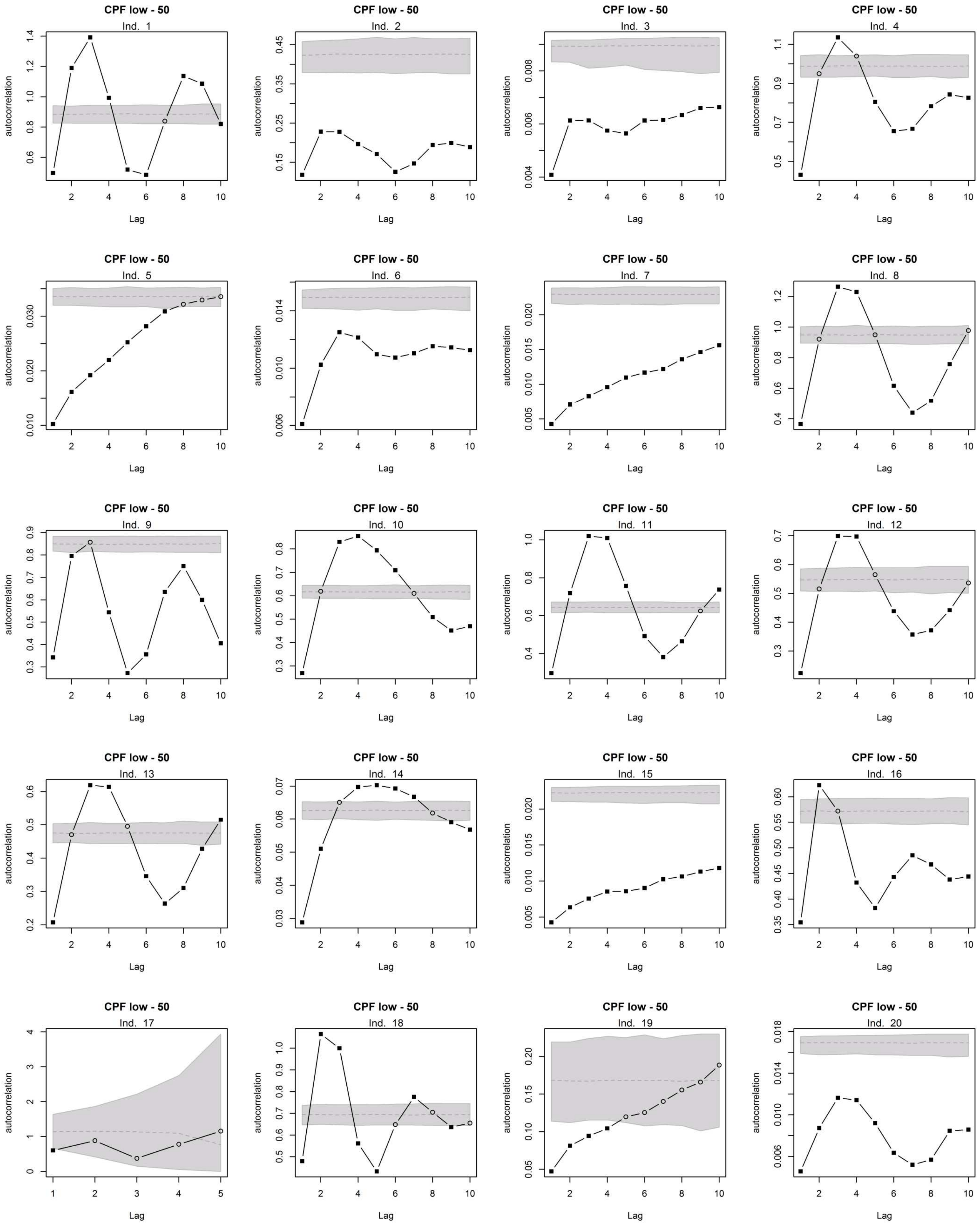

Figure S2.6g: Autocorrelation of step lengths exhibited by each observed individual over 10 lags.

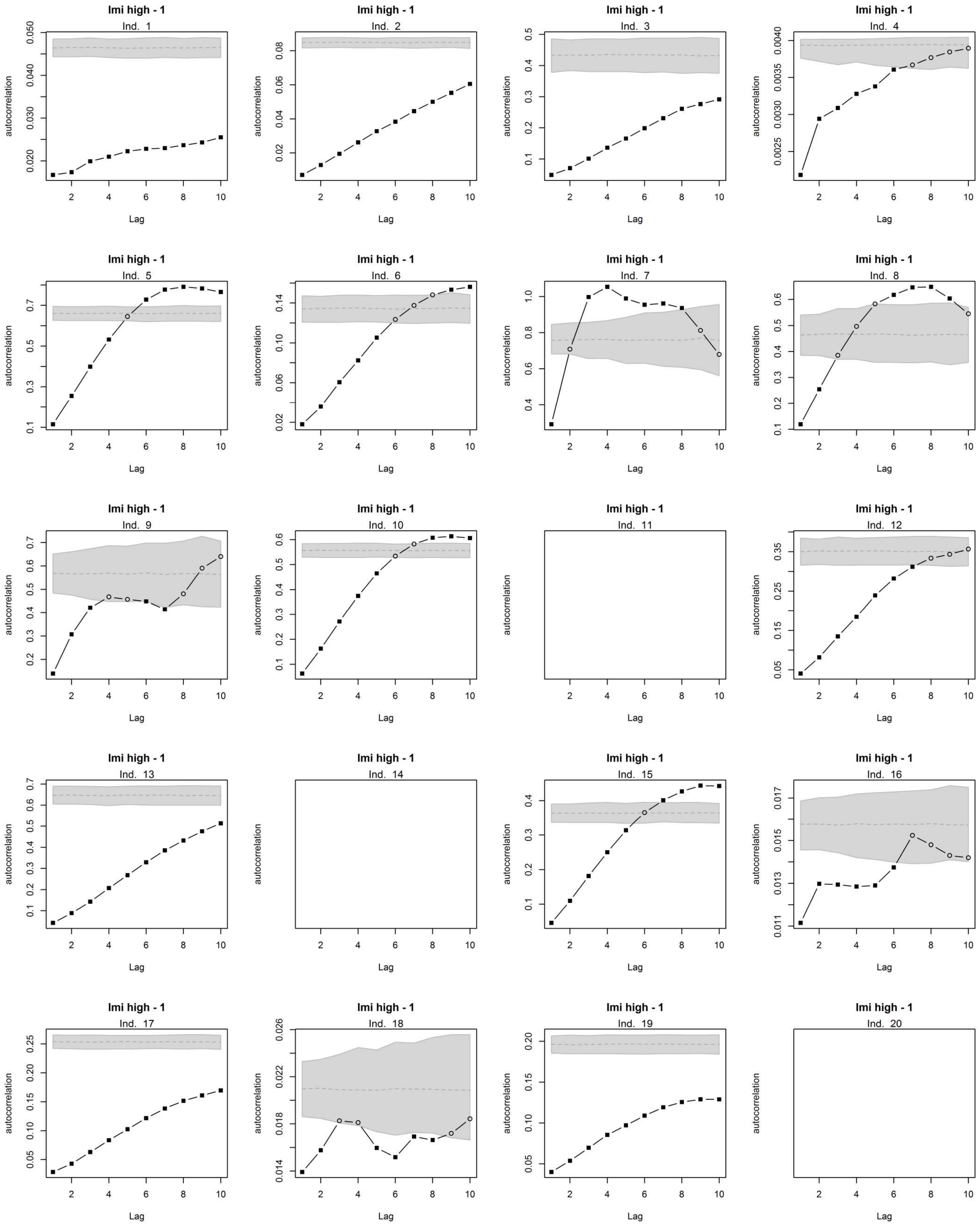

Figure S2.6h: Autocorrelation of step lengths exhibited by each observed individual over 10 lags.

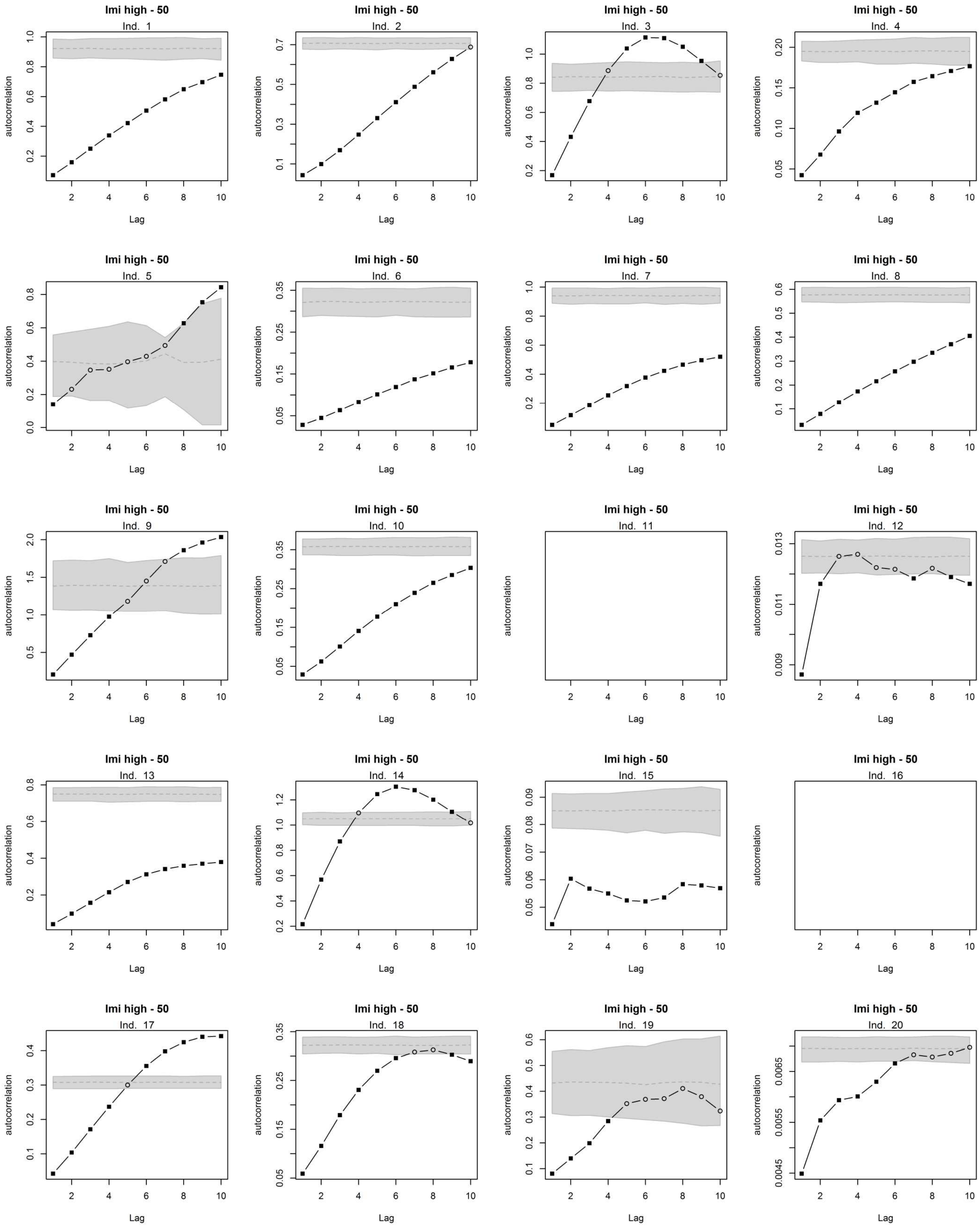

Figure S2.6i: Autocorrelation of step lengths exhibited by each observed individual over 10 lags.

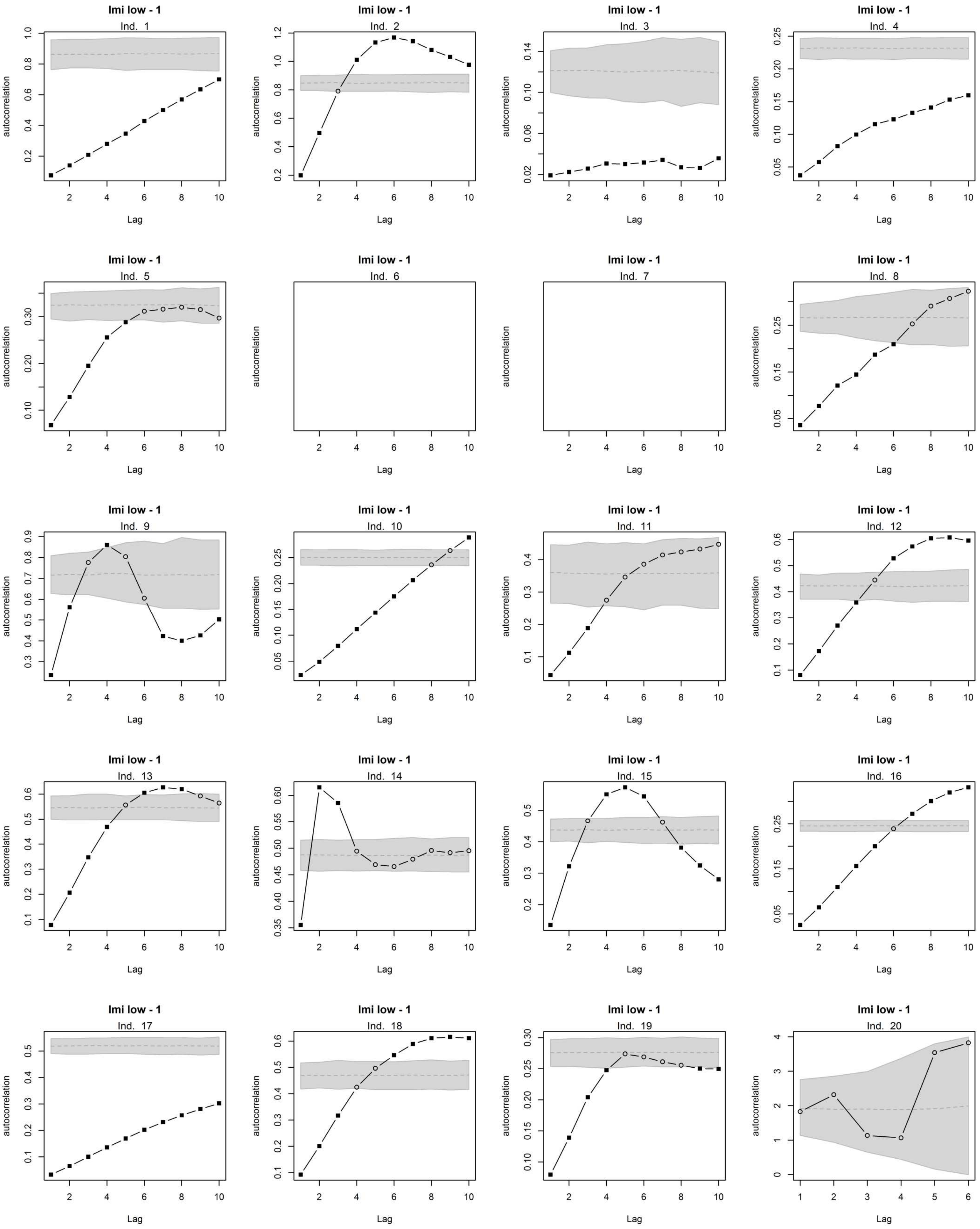

Figure S2.6j: Autocorrelation of step lengths exhibited by each observed individual over 10 lags.

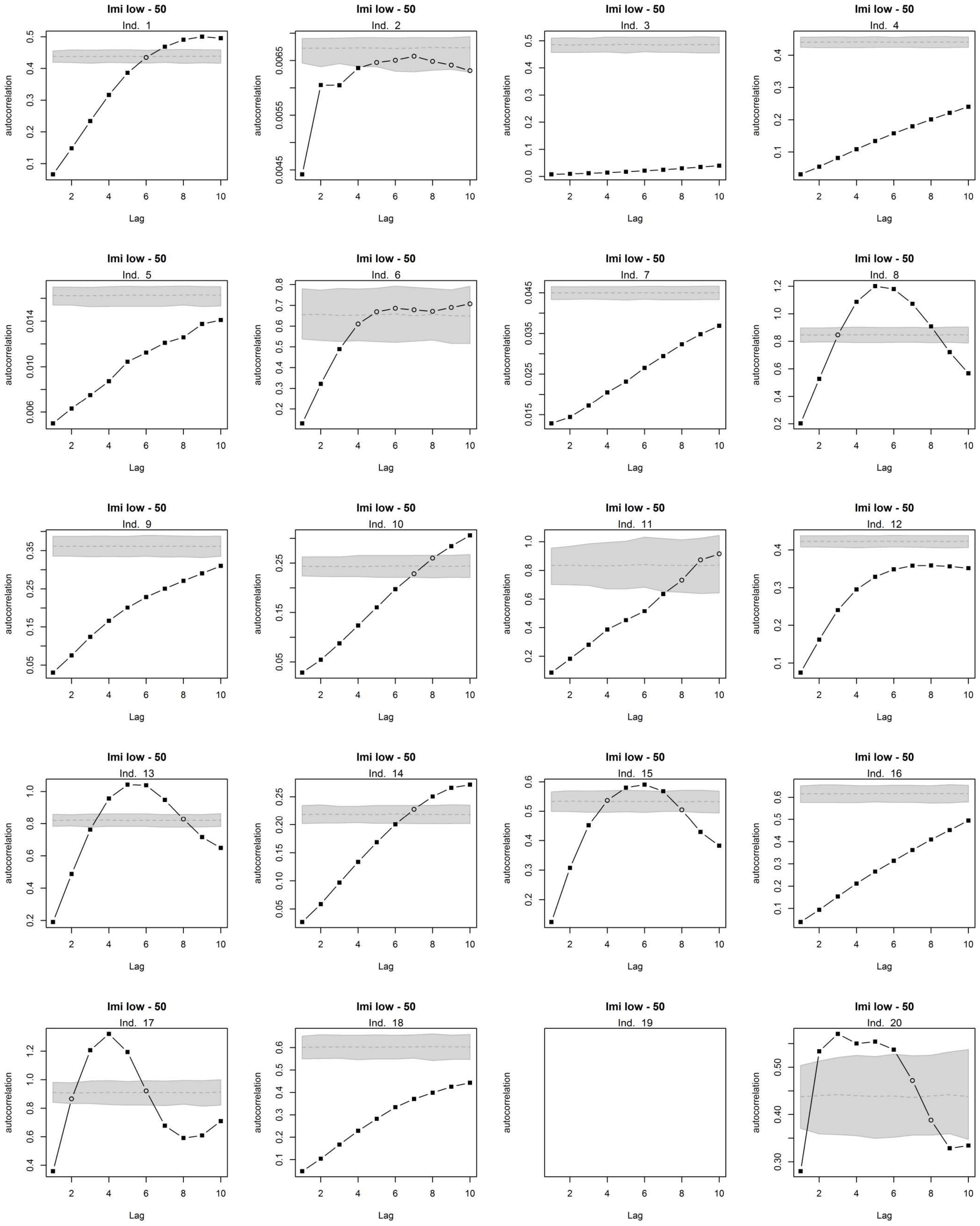

Figure S2.6k: Autocorrelation of step lengths exhibited by each observed individual over 10 lags.

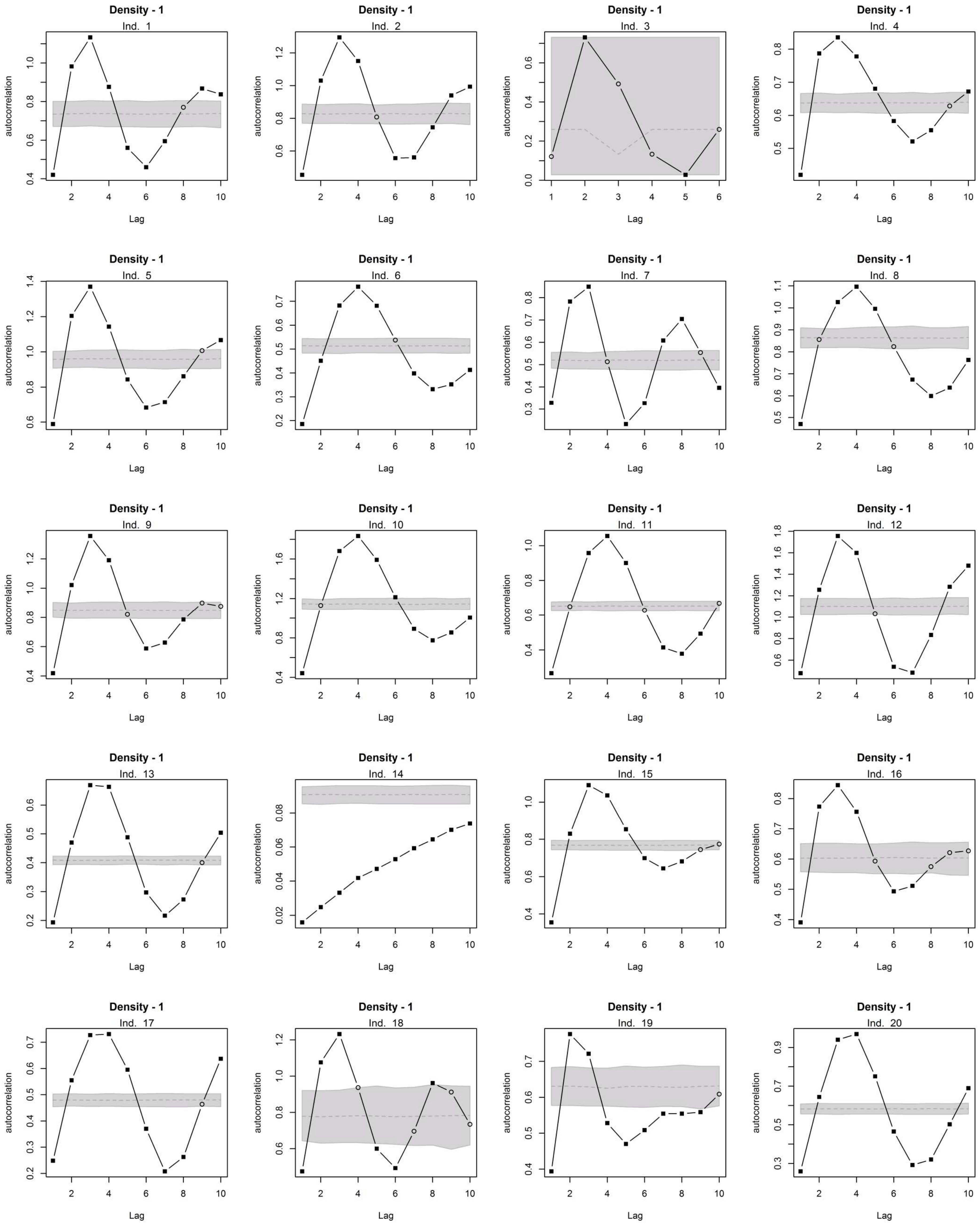

Figure S2.6l: Autocorrelation of step lengths exhibited by each observed individual over 10 lags.

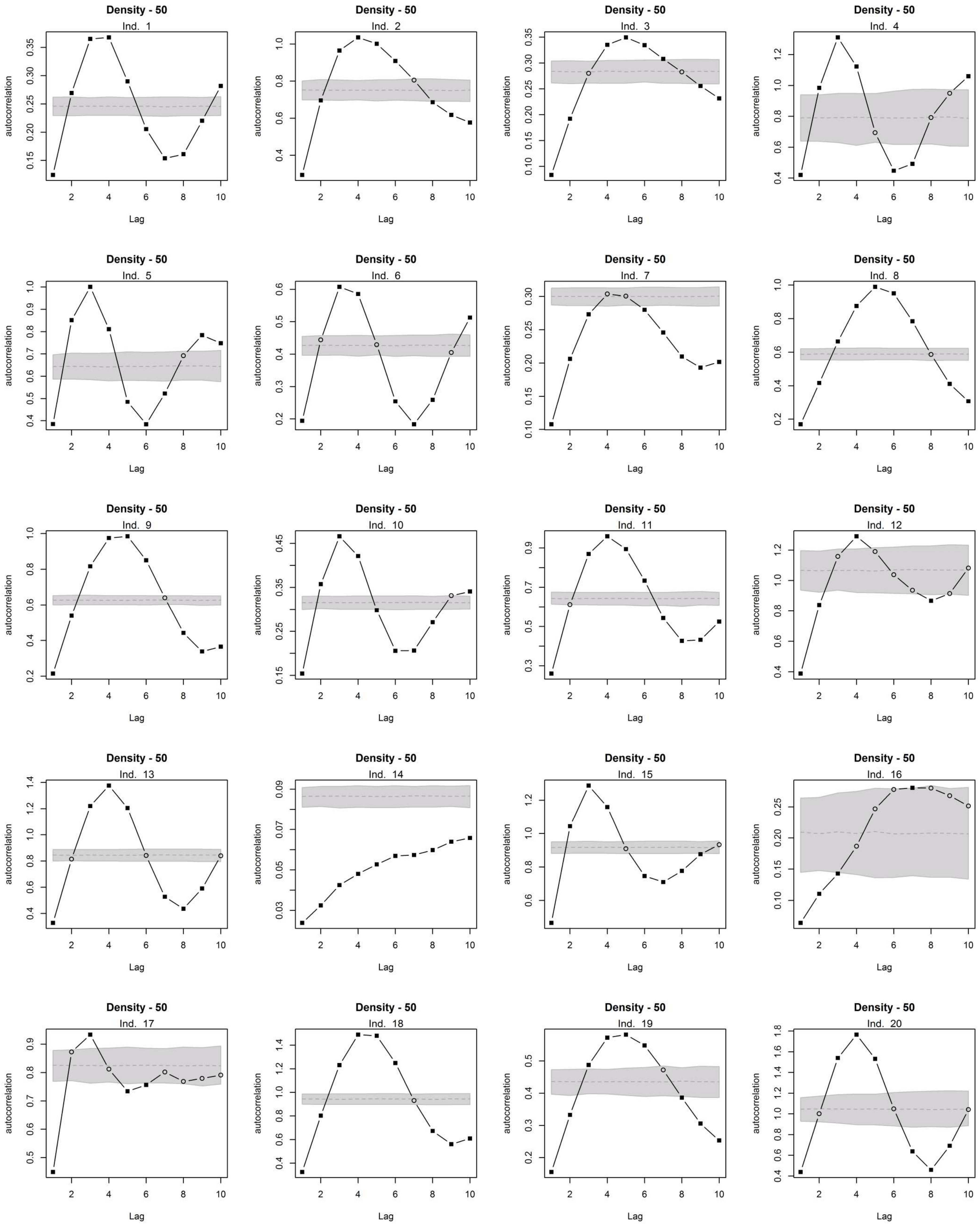

Figure S2.6m: Autocorrelation of step lengths exhibited by each observed individual over 10 lags.

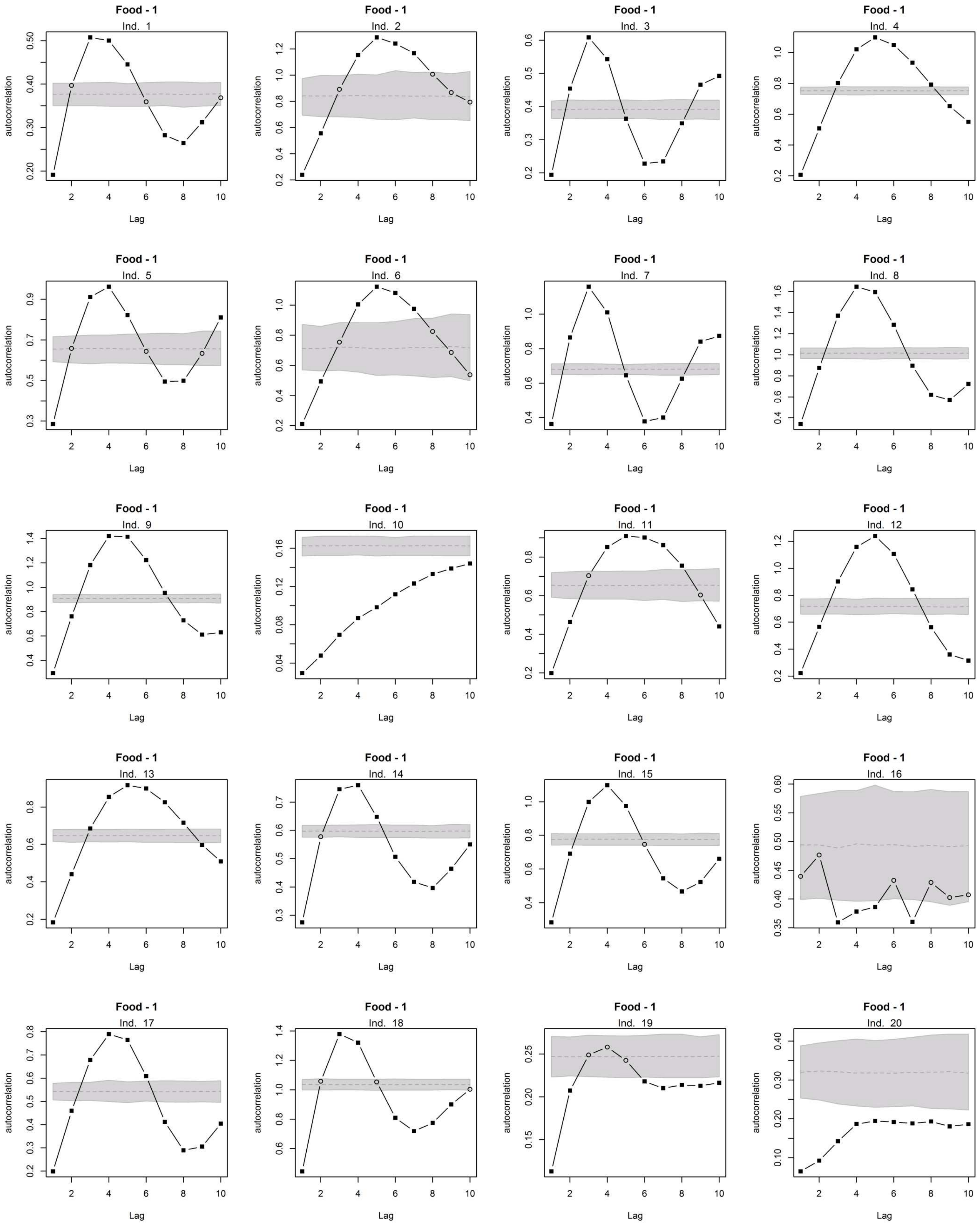

Figure S2.6n: Autocorrelation of step lengths exhibited by each observed individual over 10 lags.

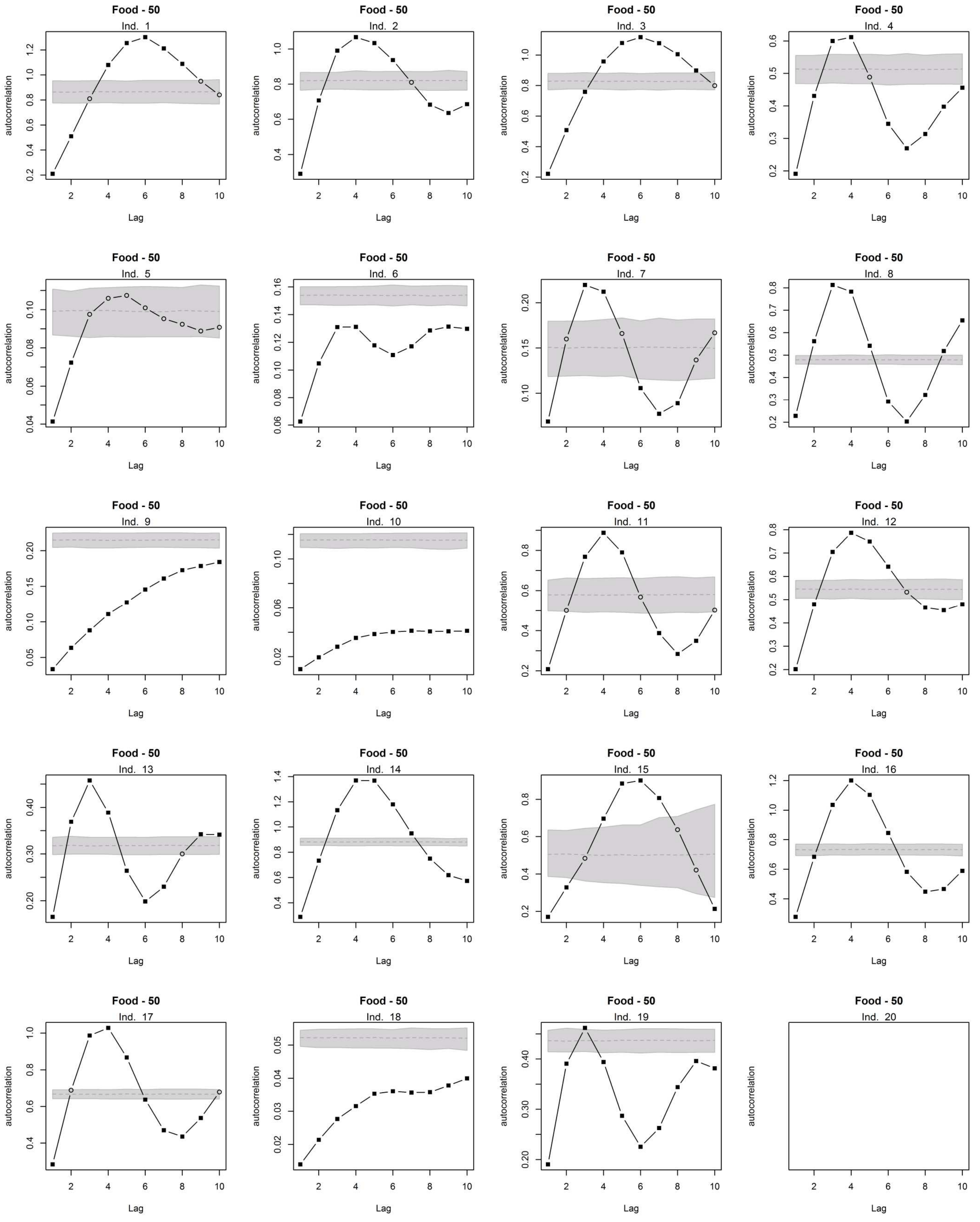

Figure S2.6o: Autocorrelation of step lengths exhibited by each observed individual over 10 lags.

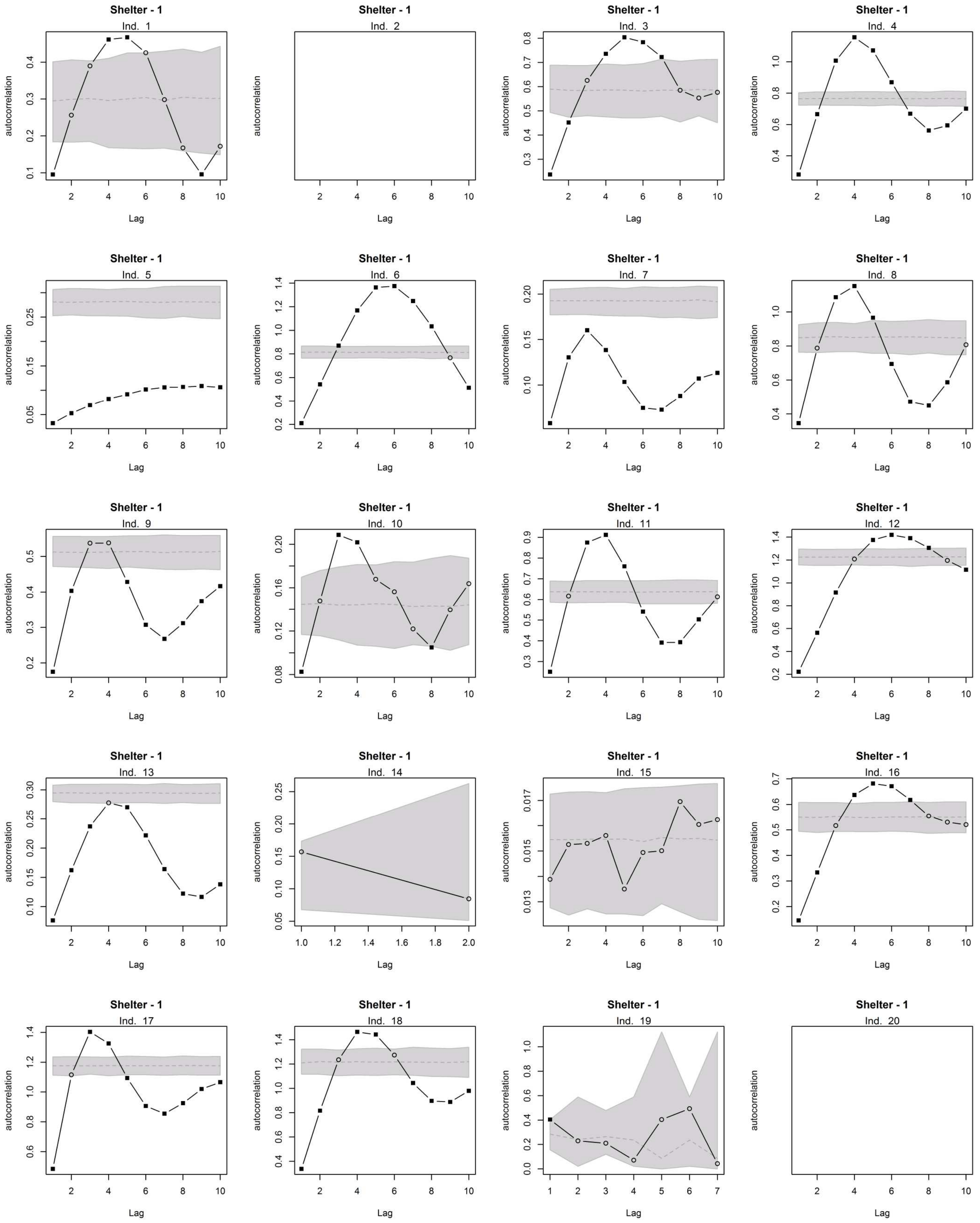

Figure S2.6p: Autocorrelation of step lengths exhibited by each observed individual over 10 lags.

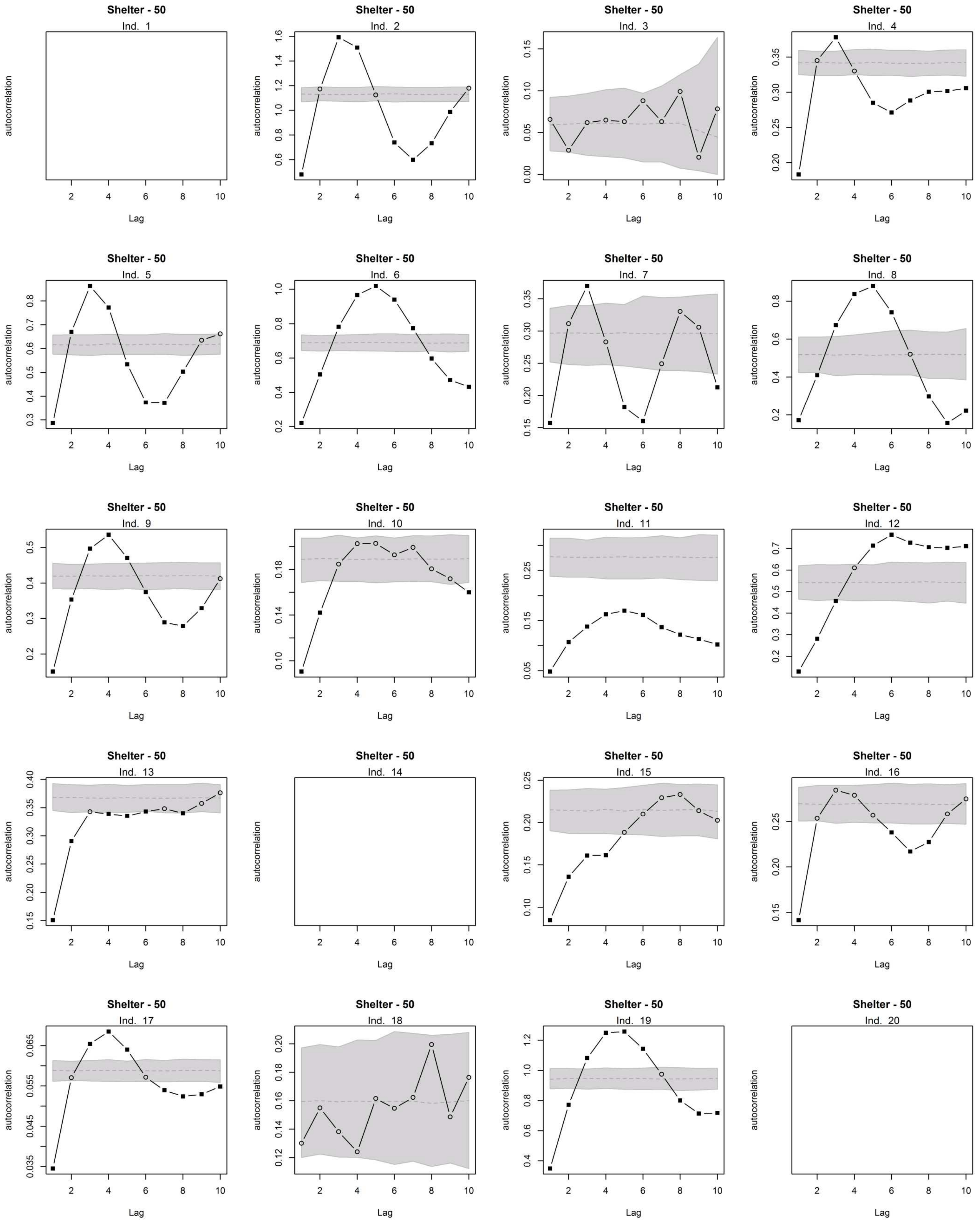

Figure S2.7a: Autocorrelation of turning angles exhibited by each observed individual over 10 lags.

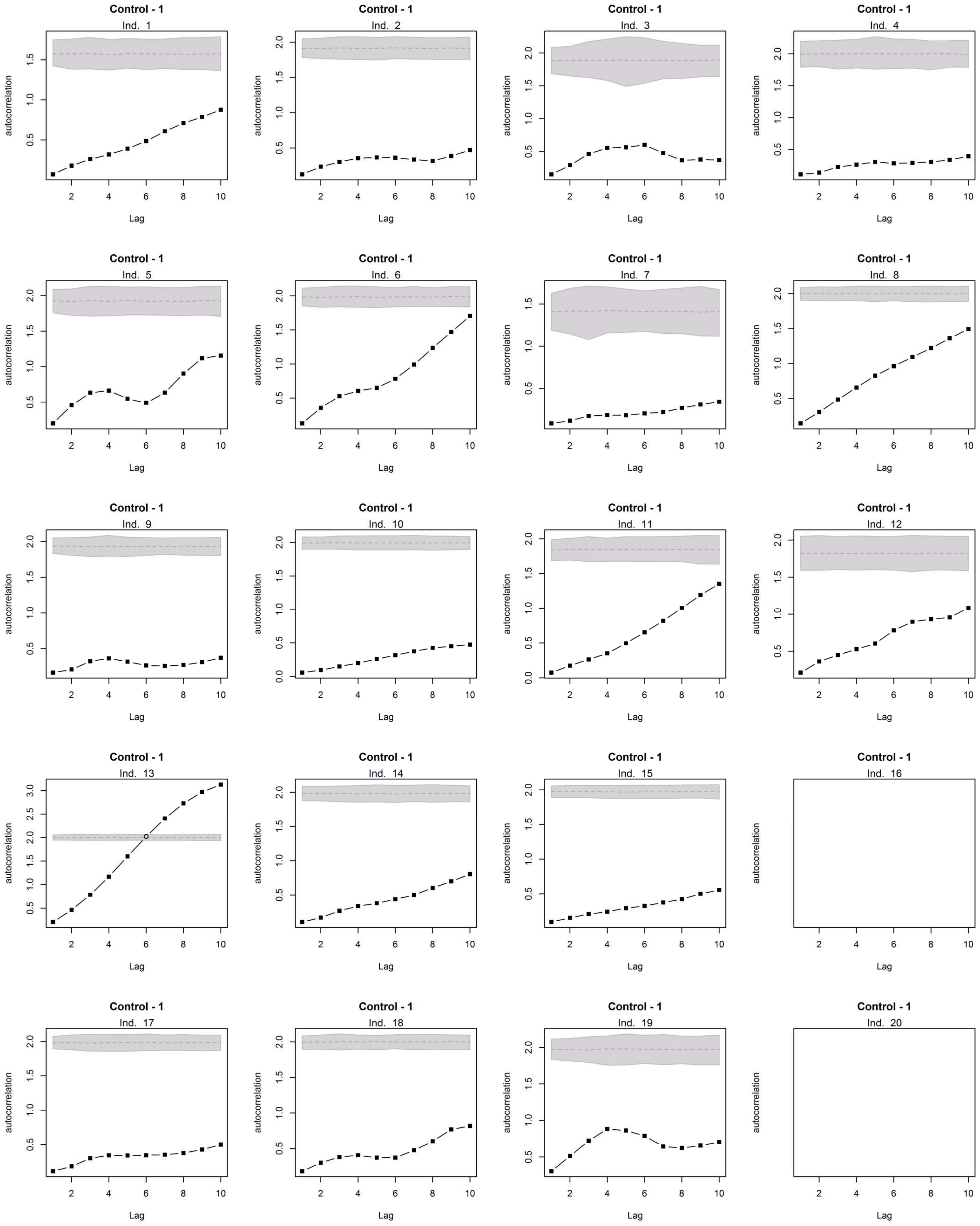

Figure S2.7b: Autocorrelation of turning angles exhibited by each observed individual over 10 lags.

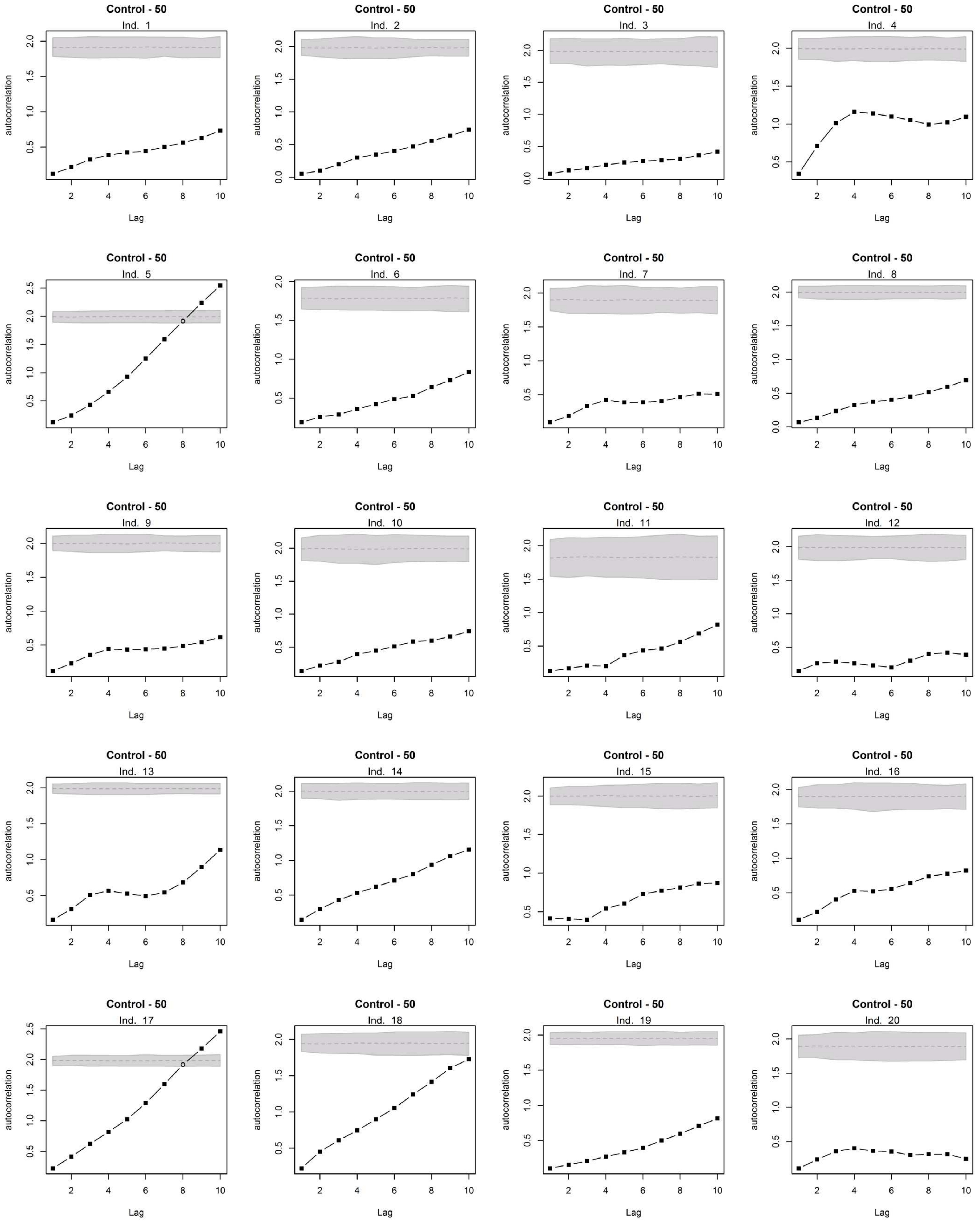

Figure S2.7c: Autocorrelation of turning angles exhibited by each observed individual over 10 lags.

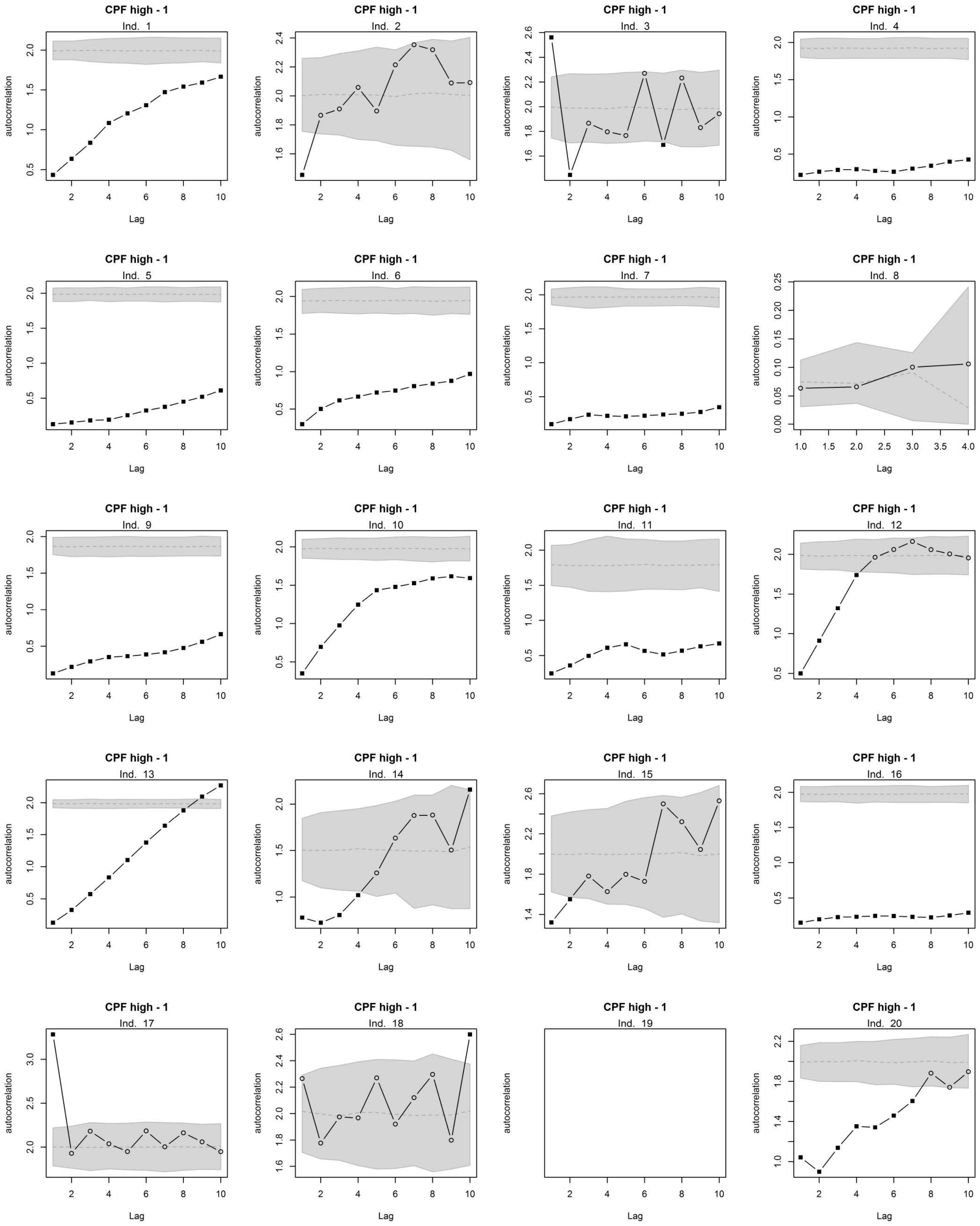

Figure S2.7d: Autocorrelation of turning angles exhibited by each observed individual over 10 lags.

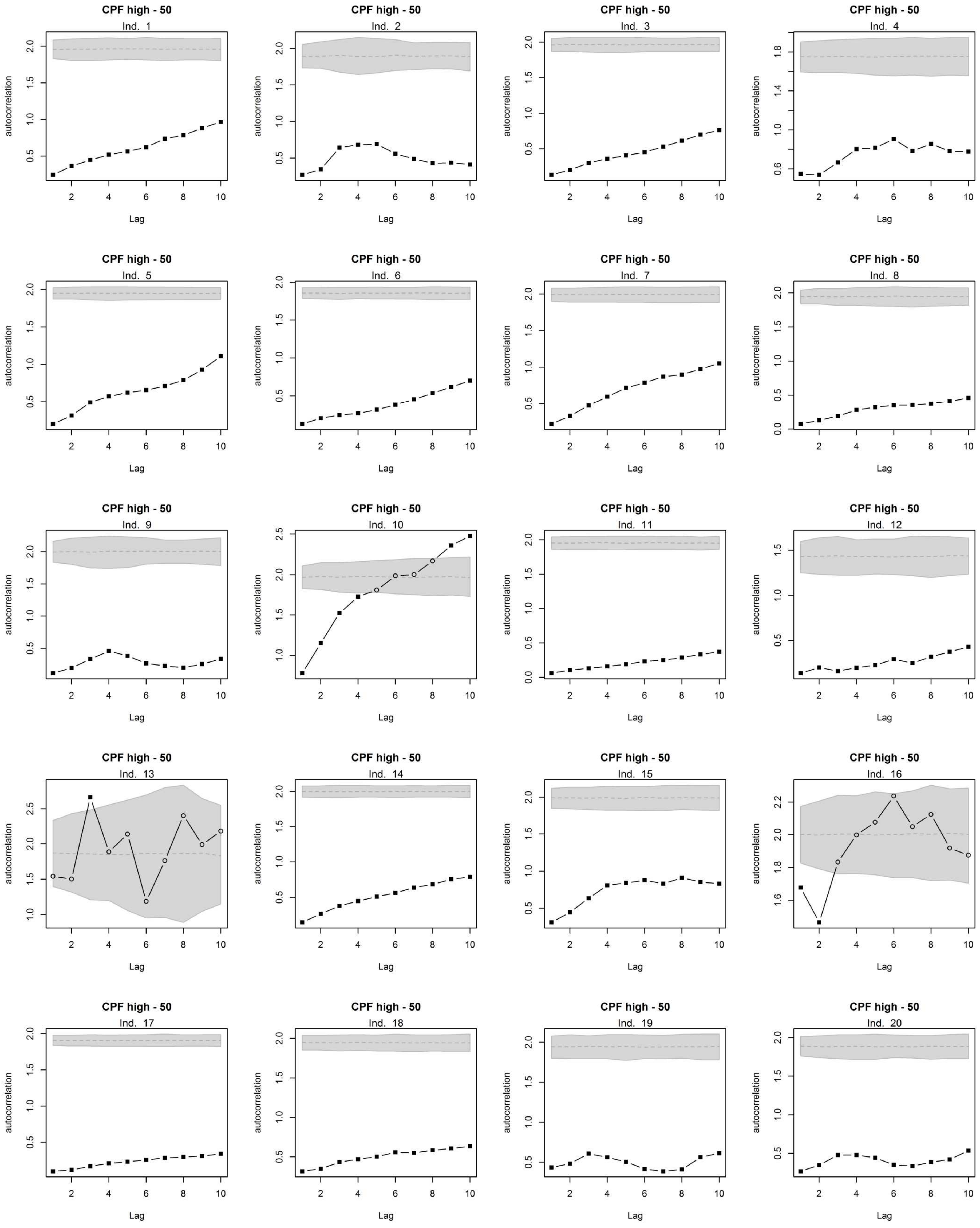

Figure S2.7e: Autocorrelation of turning angles exhibited by each observed individual over 10 lags.

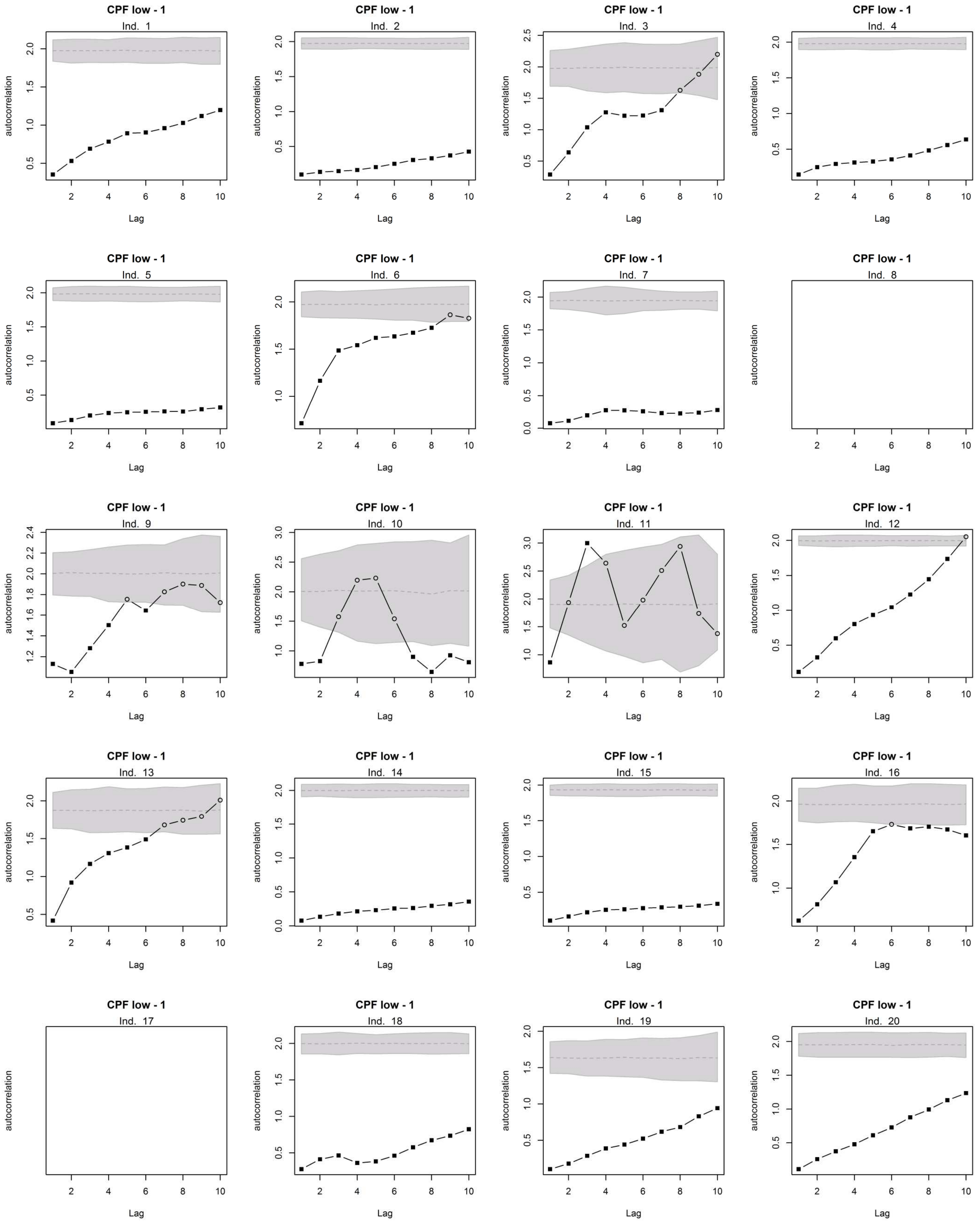

Figure S2.7f: Autocorrelation of turning angles exhibited by each observed individual over 10 lags.

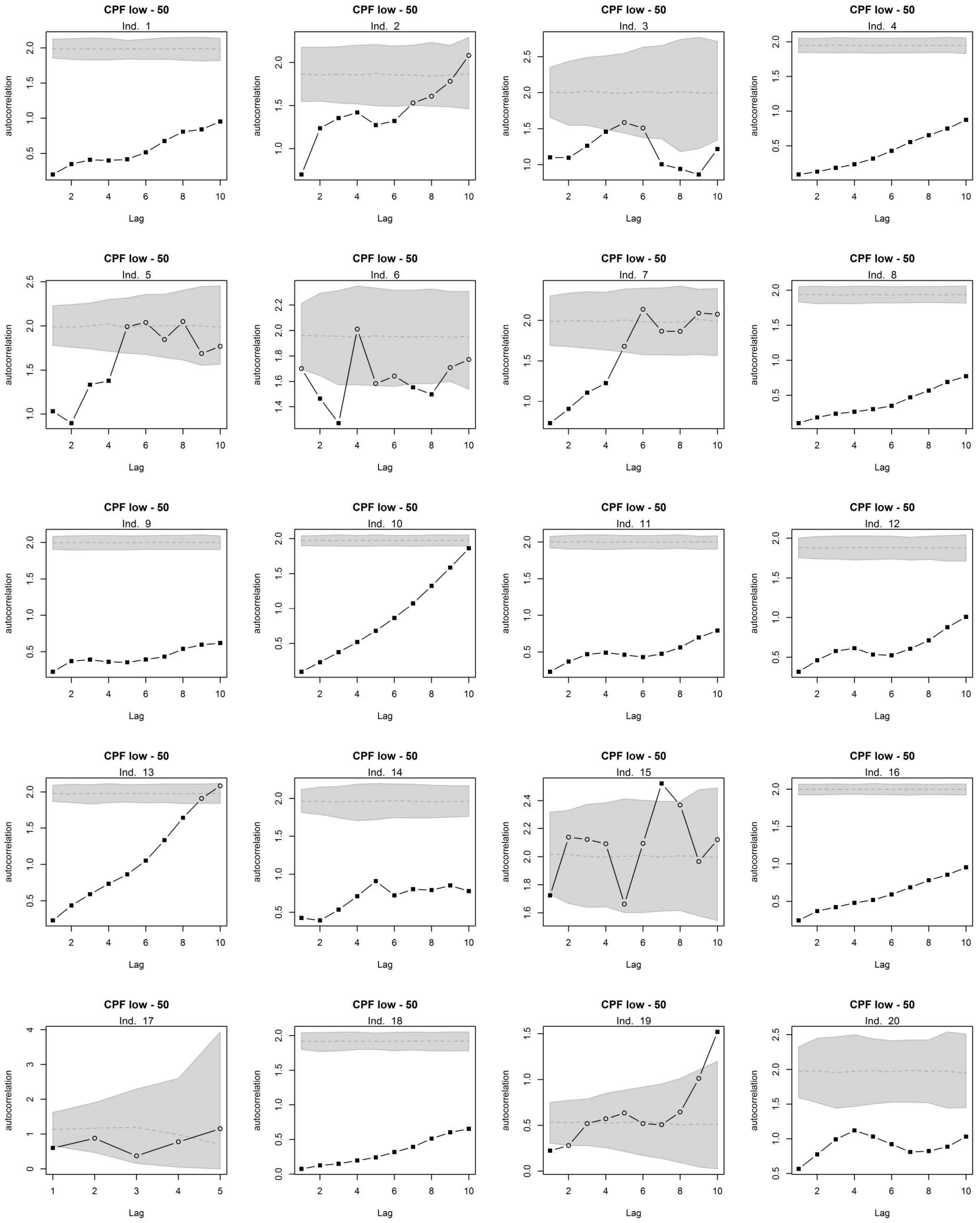

Figure S2.7g: Autocorrelation of turning angles exhibited by each observed individual over 10 lags.

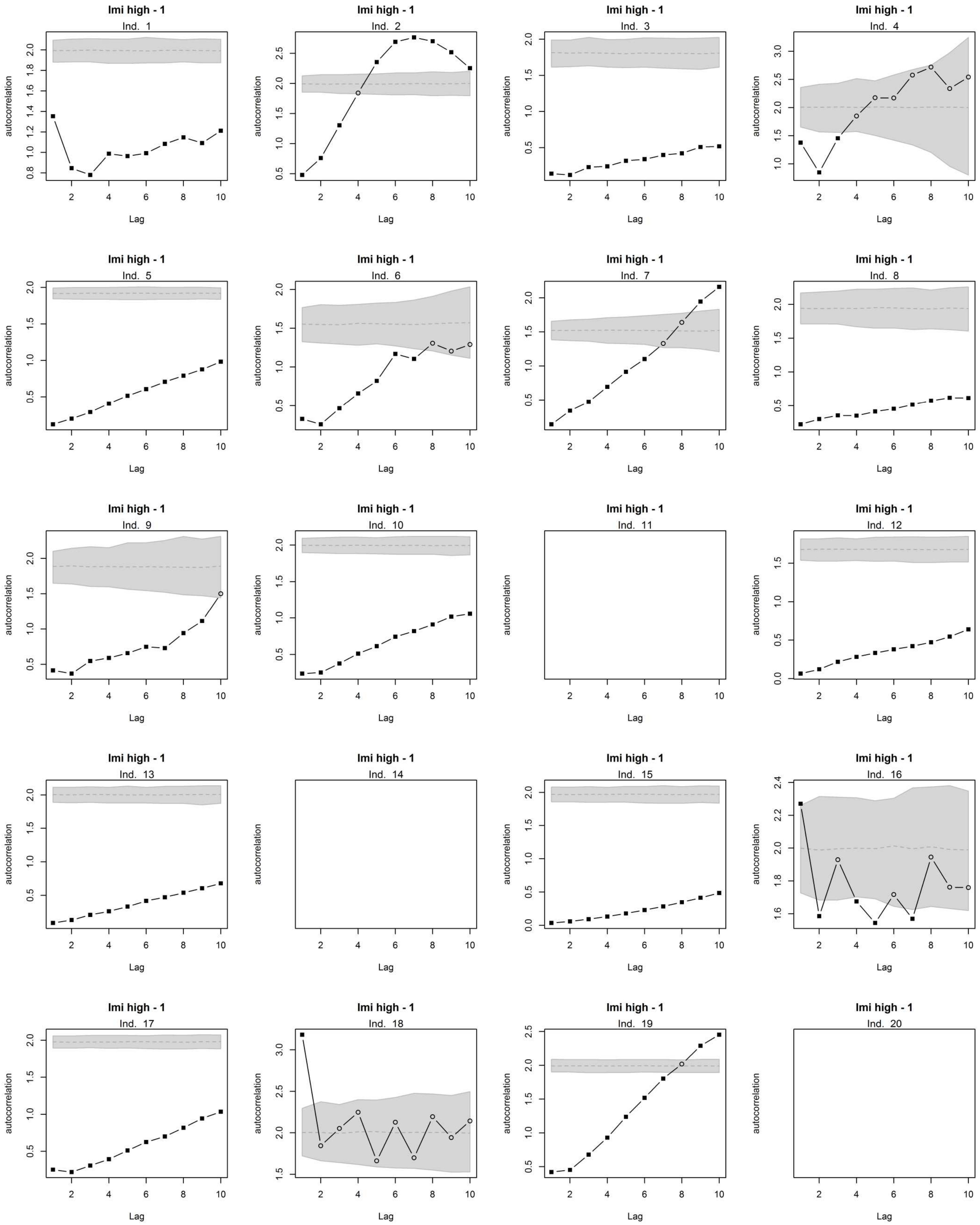

Figure S2.7h: Autocorrelation of turning angles exhibited by each observed individual over 10 lags.

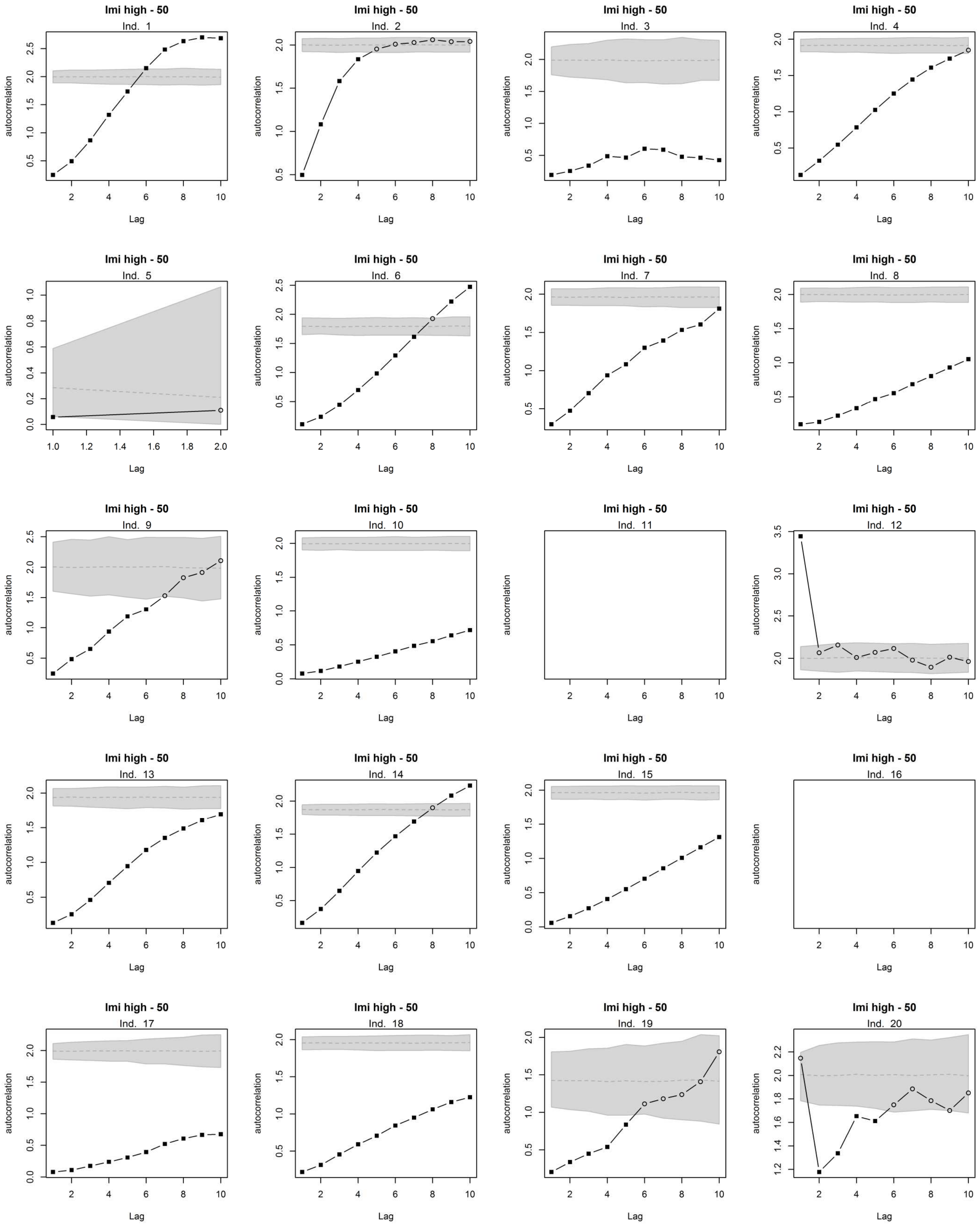

Figure S2.7i: Autocorrelation of turning angles exhibited by each observed individual over 10 lags.

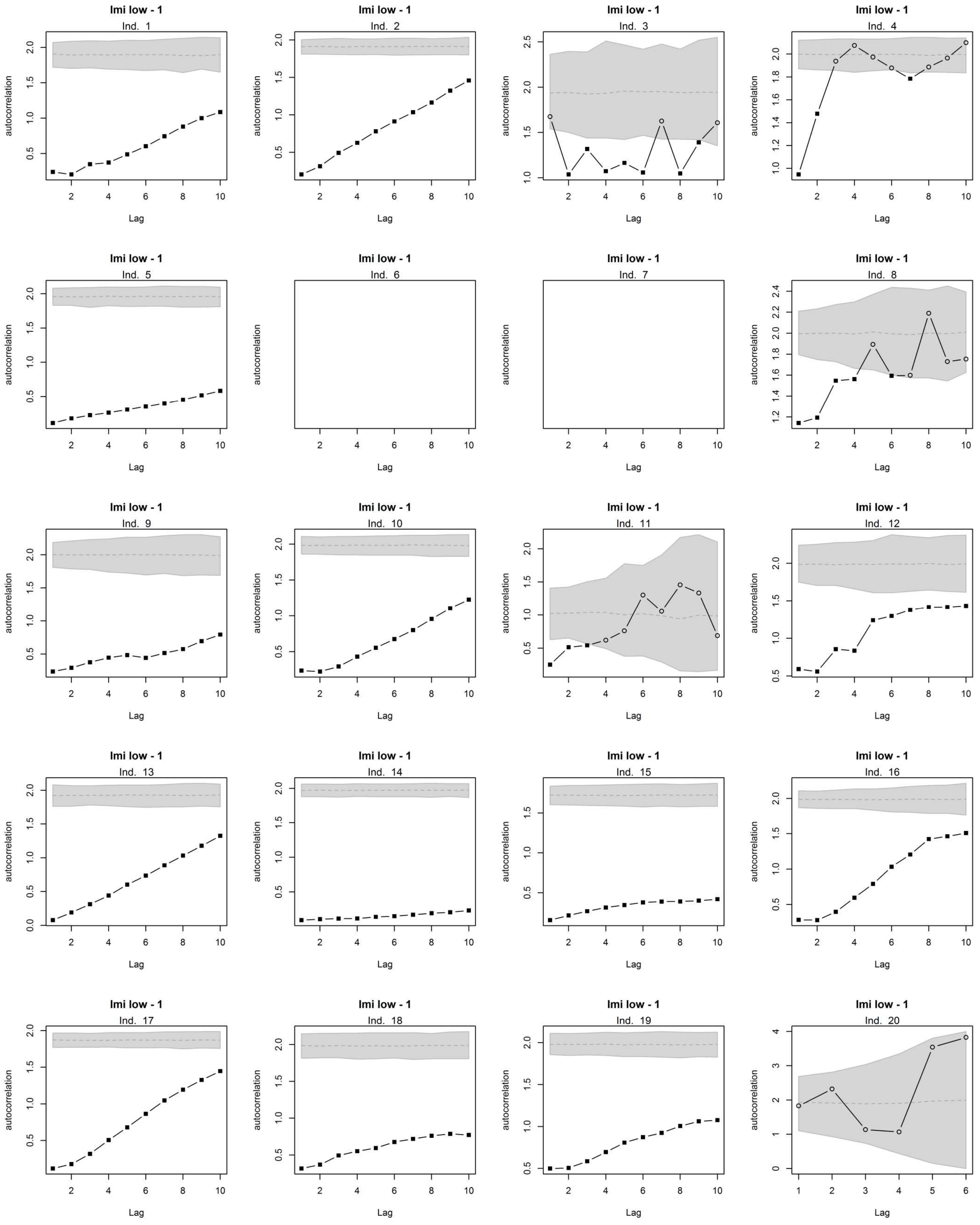

Figure S2.7j: Autocorrelation of turning angles exhibited by each observed individual over 10 lags.

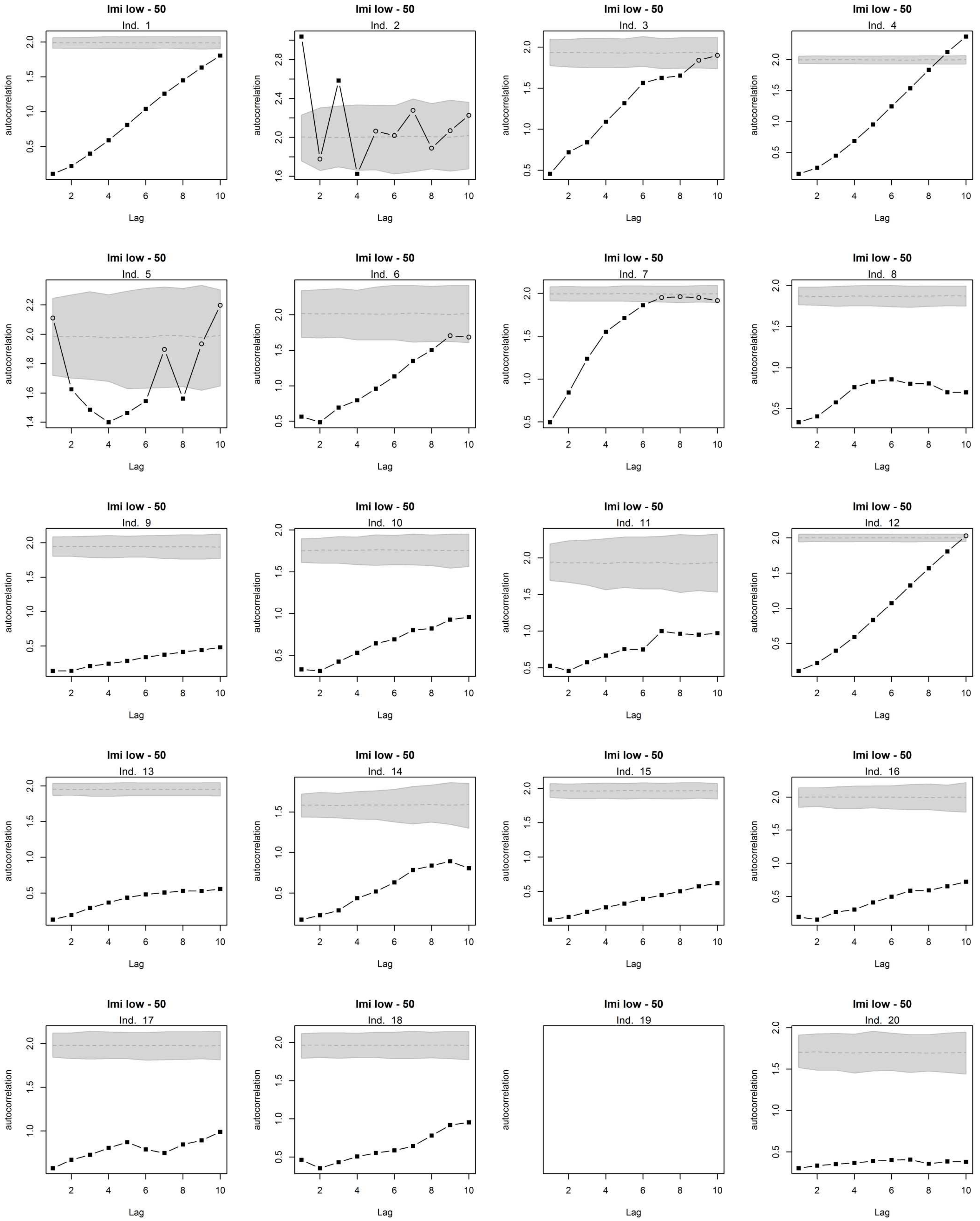

Figure S2.7k: Autocorrelation of turning angles exhibited by each observed individual over 10 lags.

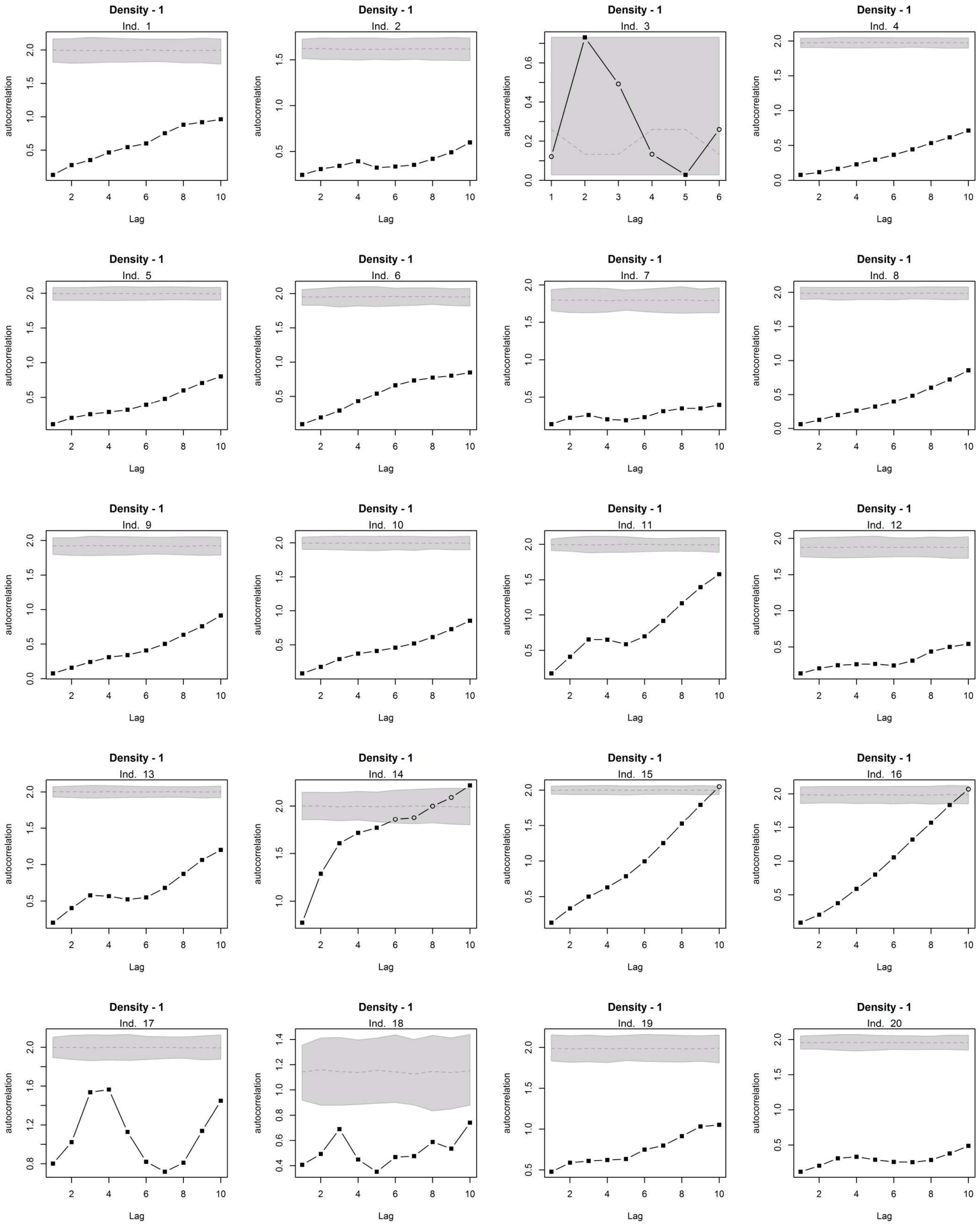

Figure S2.7l: Autocorrelation of turning angles exhibited by each observed individual over 10 lags.

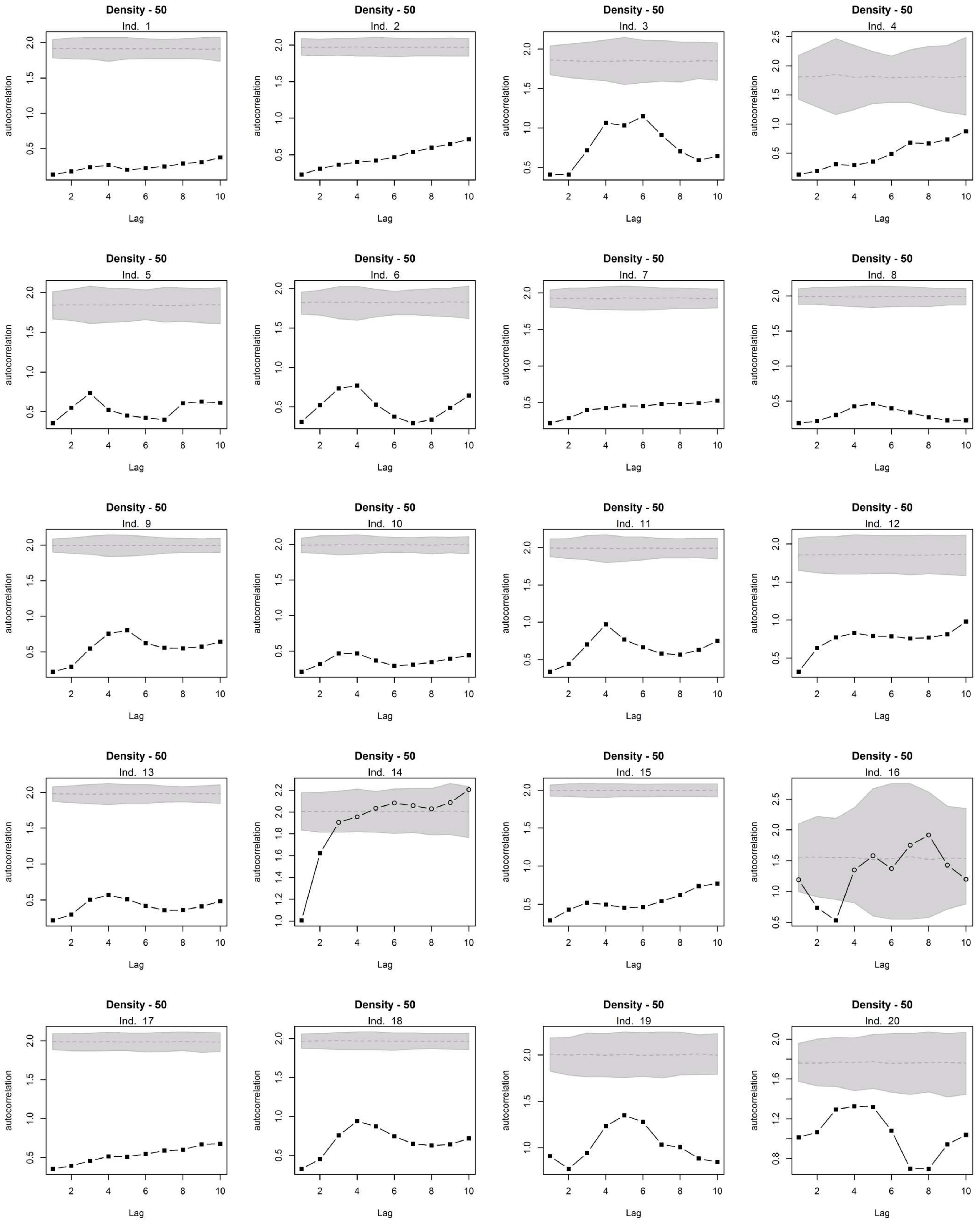

Figure S2.7m: Autocorrelation of turning angles exhibited by each observed individual over 10 lags.

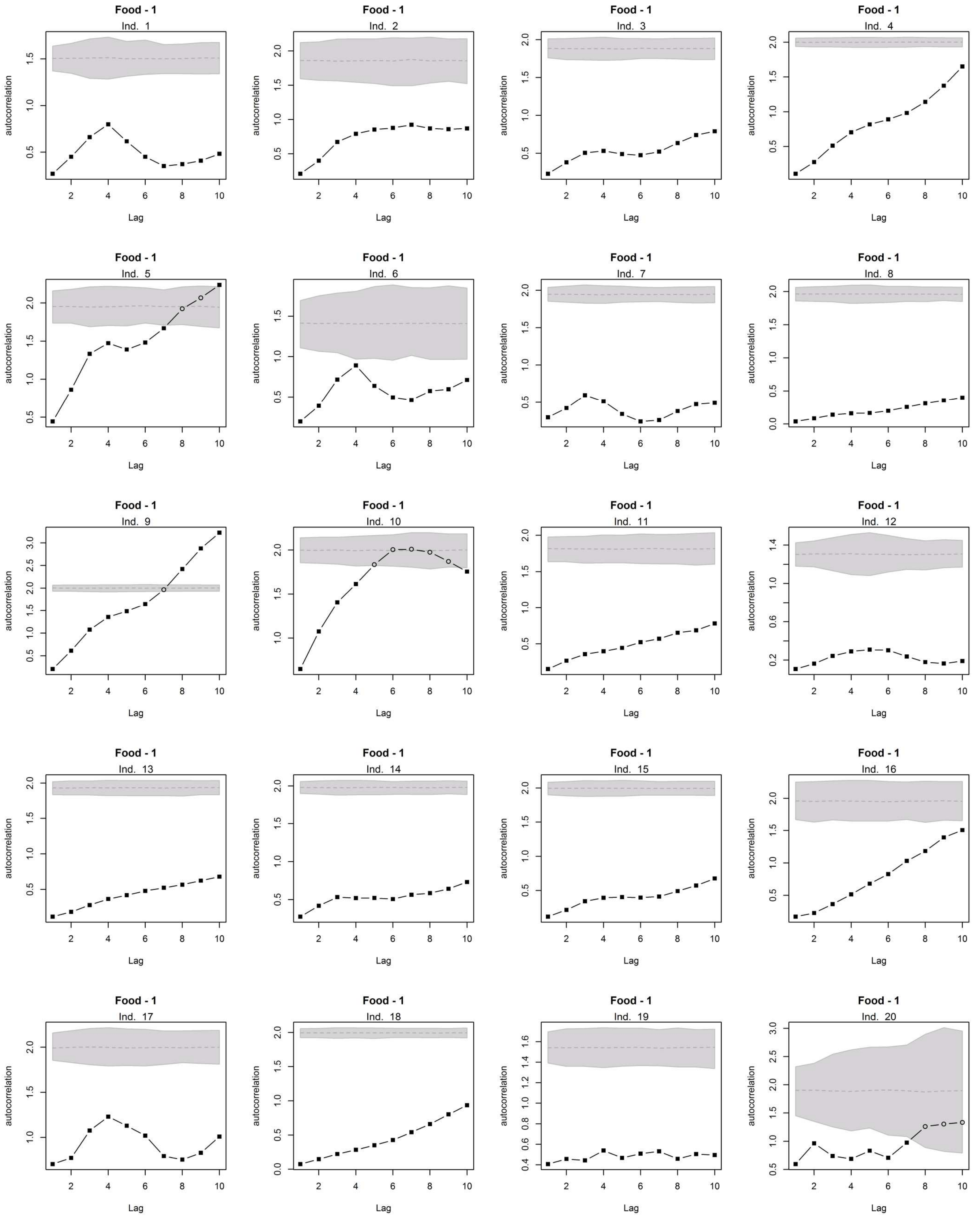

Figure S2.7n: Autocorrelation of turning angles exhibited by each observed individual over 10 lags.

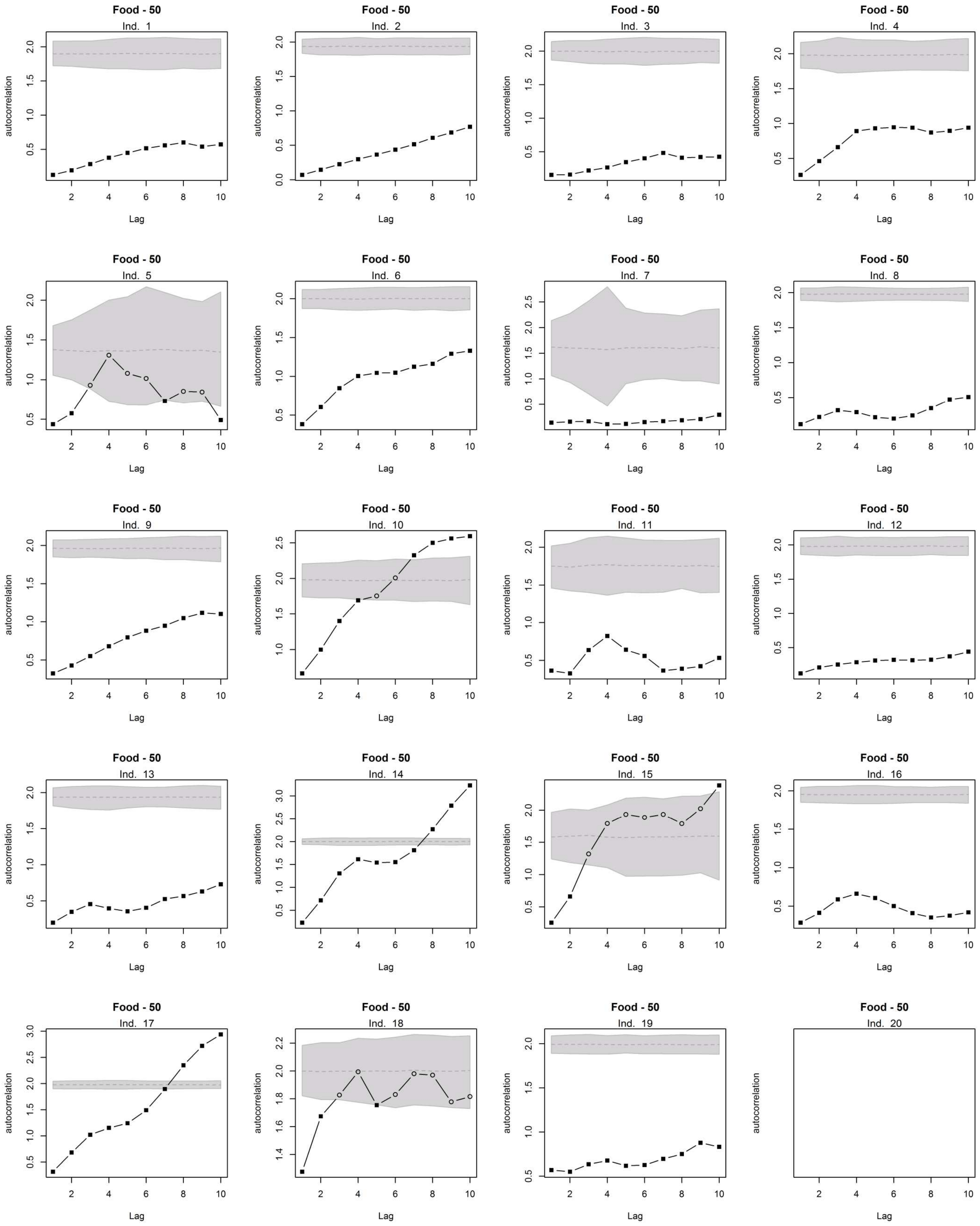

Figure S2.7o: Autocorrelation of turning angles exhibited by each observed individual over 10 lags.

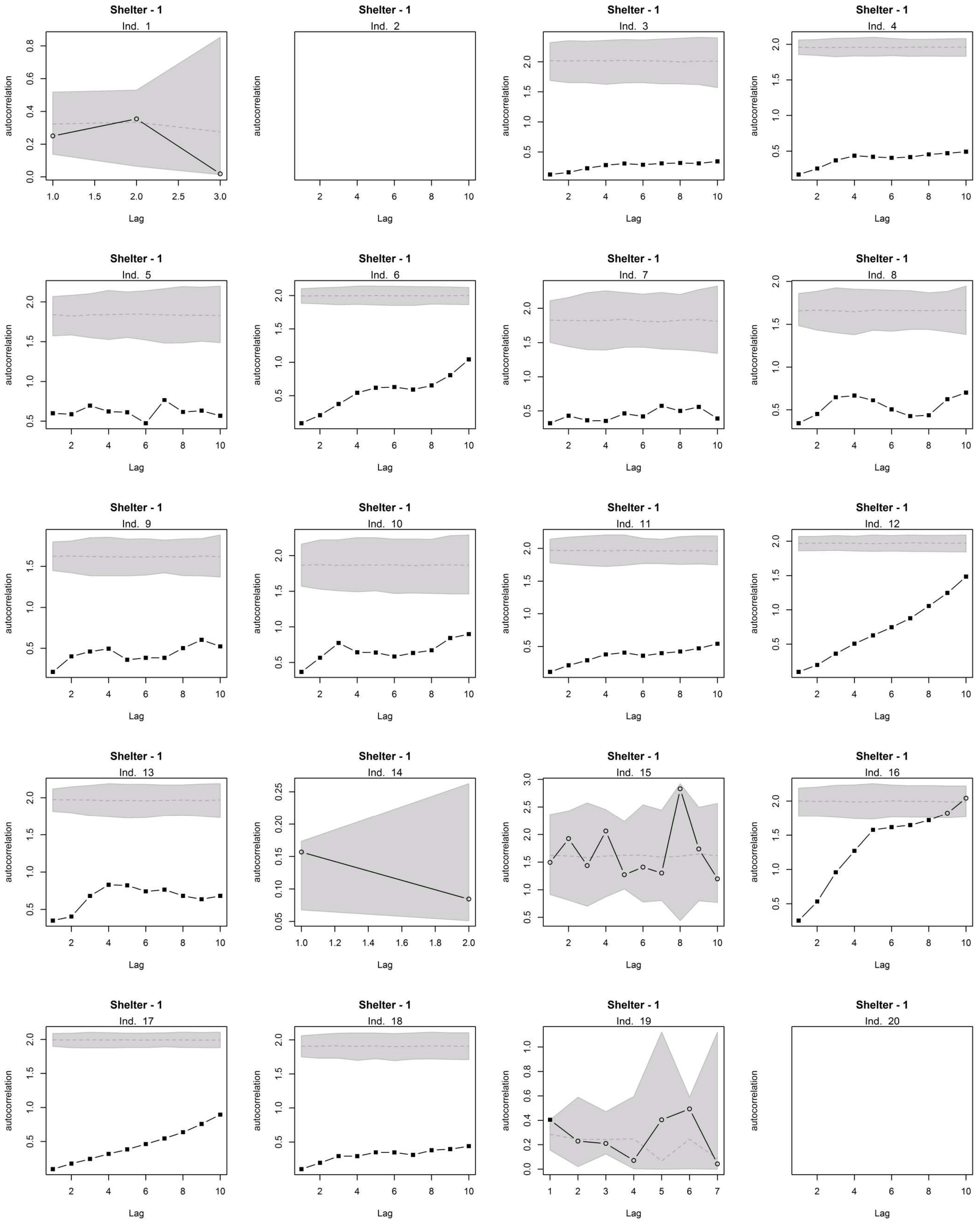

Figure S2.7p: Autocorrelation of turning angles exhibited by each observed individual over 10 lags.

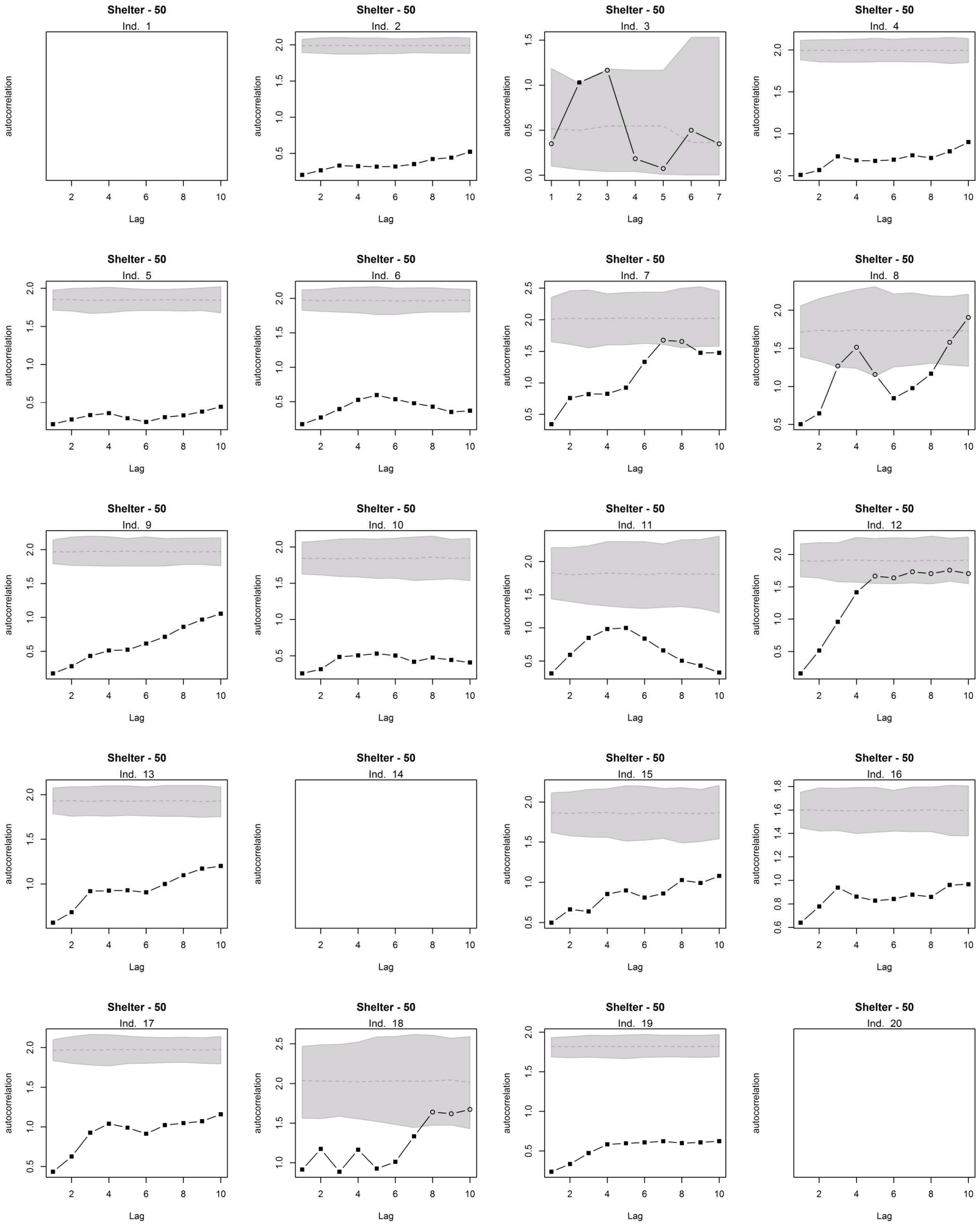

Supplement: Supplementary file 2 — Online Resource 2: Detailed results of the movement behaviour study. (PDF 10326 kb) [file 10646_2016_1686_MOESM2_ESM.pdf]
